# Synthesis and photoinduced behavior of DPP-anchored nitronyl nitroxides: a multifaceted approach†

**DOI:** 10.1039/d4ra00916a

**Published:** 2024-02-19

**Authors:** Evgeny Tretyakov, Dmitry Gorbunov, Nina Gritsan, Ashok Keerthi, Martin Baumgarten, Dieter Schollmeyer, Mikhail Ivanov, Anna Sergeeva, Matvey Fedin

**Affiliations:** a N.D. Zelinsky Institute of Organic Chemistry, Russian Academy of Sciences Leninsky Ave. 47 Moscow 119991 Russian Federation tretyakov@ioc.ac.ru; b V.V. Voevodsky Institute of Chemical Kinetics and Combustion 3 Institutskaya Str. Novosibirsk 630090 Russian Federation nina.gritsan@gmail.com; c Department of Chemistry, School of Natural Sciences, The University of Manchester Oxford Road M13 9PL UK; d Max Planck Institute for Polymer Research Ackermannweg 10 Mainz D-55128 Germany; e Johannes Gutenberg-University Mainz Duesbergweg 10-14 55128 Mainz Germany; f International Tomography Center 3a Institutskaya Str. Novosibirsk 630090 Russian Federation

## Abstract

Understanding and controlling spin dynamics in organic dyes is of significant scientific and technological interest. The investigation of 2,5-dihydropyrrolo[4,3-*c*]pyrrolo-1,4-dione derivatives (DPPs), one of the most widely used dyes in many fields, has so far been limited to closed-shell molecules. We present a comprehensive joint experimental and computational study of DPP derivatives covalently linked to two nitronyl nitroxide radicals (DPP^Th^-NN_2_). Synthesis, single crystal X-ray diffraction study, photophysical properties, magnetic properties established using steady-state and pulse EPR, fast spin dynamics, and computational modelling using density functional theory and *ab initio* methods of electronic structure and spectroscopic properties of DPP^Th^-NN_2_ are presented. The single-crystal X-ray diffraction analysis of DPP^Th^-NN_2_ and computational modeling of its electronic structure suggest that effective conjugation along the backbone leads to noticeable spin-polarization transfer. Calculations using *ab initio* methods predict a weak exchange interaction of radical centers through a singlet ground state of DPP^Th^ with a small singlet–triplet splitting (Δ*E*_ST_) of about 25 cm^−1^ (∼0.07 kcal mol^−1^). In turn, a strong ferromagnetic exchange interaction between the triplet state of DPP^Th^ chromophore and nitronyl nitroxides (with *J* ∼ 250 cm^−1^) was predicted.

## Abstract 2

Understanding and controlling spin dynamics in organic dyes is of significant scientific and technological interest.

## Graphical abstract

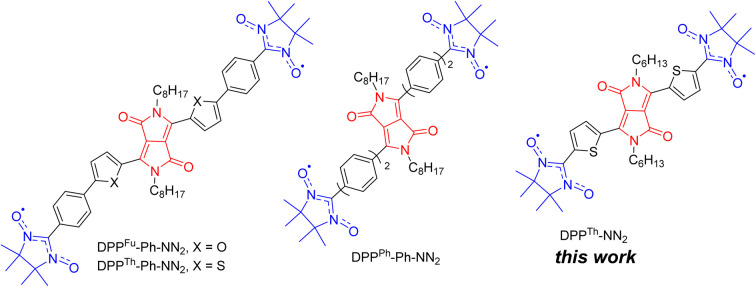

## Introduction

Discovered in the early 1970s,^1^ diketopyrrolopyrroles (2,5-dihydropyrrolo[4,3-*c*]pyrrolo-1,4-diones, DPPs) remain one of the most widely used dyes, finding application in many areas of high technology. Numerous studies have been devoted to the development of DPP derivatives as high-performance materials for use in various applications such as high-performance pigments, organic field-effect transistors, bulk-heterojunction solar cells, dye-sensitized solar cells, organic light-emitting diodes, fluorescence imaging, and many other fields.^2^ Despite the significant progress achieved by researchers in the exploration of DPP-based compounds, only recently have their conjugated derivatives substituted with stable radical groups been obtained. Single crystal X-ray data on a DPP derivative bearing radical groups, namely, bis(thiophen-2-yl)-2,5-dihydropyrrolo[3,4-*c*]pyrrole-1,4-dione with terminal nitronyl nitroxide groups (DPP^Th^-NN_2_) were first reported in 2020.^3^

**Fig. 1:**
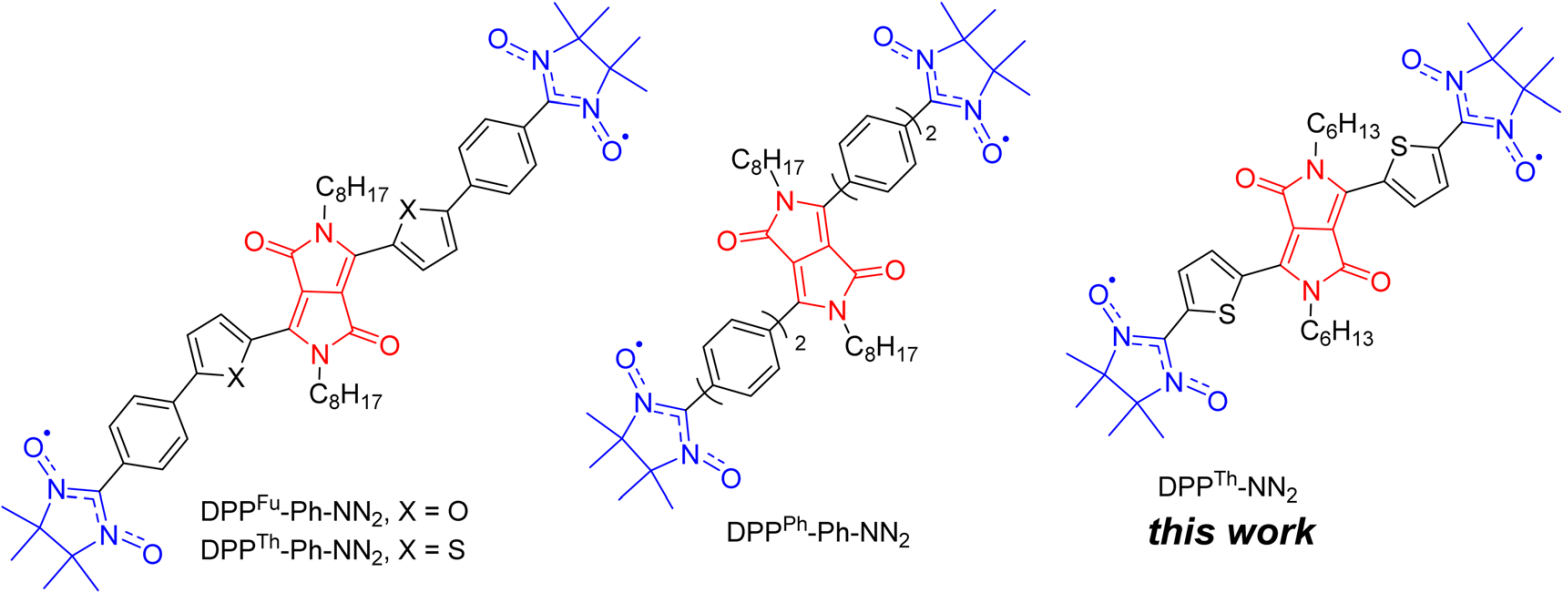
Chemical structures of DPP-linked nitronyl-nitroxide diradicals: DPP^Th^-NN_2_, DPP^Th^-Ph-NN_2_, DPP^Fu^-Ph-NN_2_, and DPP^Ph^-Ph-NN_2_.

Later, nitronyl-nitroxide diradicals with extended DPP-containing linkers (DPP^Th^-Ph-NN_2_, DPP^Fu^-Ph-NN_2_, and DPP^Ph^-Ph-NN_2_, Fig. 1) were obtained and partially characterized.^4^ In addition to the listed diradicals (Fig. 1), perylene bisimide (PBI) and iso-indigo (IIn) conjugated diradicals were also synthesized.^4^ Although all diradicals retain the intrinsic optical properties of the dyes or, more specifically, pi-conjugated chromophore, at the same time they exhibit an indirect spin coupling between two distant paramagnetic centres. Room temperature EPR data^4^ provided only nearly identical 9-line spectra, demonstrating that the exchange coupling parameter *J* is much larger than twice the hyperfine coupling constant. The distances between the radical units were very large (C2–C2′ distance in the range 2.2–2.4 nm for DPP-based diradicals) due to the additional phenyl rings connecting the NN moieties to the DPP core.^4^ To date, comprehensive temperature-dependent studies of liquid and frozen solutions *via* EPR spectroscopy, as well as magnetization measurements of polycrystalline powder, have not been conducted. These analyses are crucial for a deeper understanding and clear differentiation of the magnetic properties of such diradicals. Earlier, Matsuda *et al.* demonstrated the exponential distance dependence of the exchange interaction parameter over extended phenylacetylene bridged NN biradicals.^5^ Their findings indicate that the EPR spectra of the examined diradicals in liquid phase will display a consistent nine-line pattern (indicative of strong exchange) when the distance between the C2 atoms of the NN moieties ranges from 0.7 to 2.7 nm. This observation is coupled with significant variations (spanning orders of magnitude) in the exchange coupling parameters, highlighting the sensitivity of magnetic interactions to molecular spacing within these systems.

Thus, the magnetic properties of DPP-based diradicals require much more in-depth studies. Since DPP derivatives have found applications in the solar cells, OLEDs, fluorescence imaging, *etc.*,^2*d*–*g*^ information available about their electronic absorption spectra is insufficient to understand their excitation relaxation dynamics which is very important to broaden the scope of these molecules in respective fields. Our study was undertaken with the aim of quantitatively elucidating the magnetic properties of the DPP^Th^-NN_2_ diradical. Continuous-wave and time-resolved EPR spectra were recorded, and exchange and dipole–dipole interactions were theoretically assessed not only in the ground state of DPP linker, but also in the case of its triplet excitation. Furthermore, we tried to resolve the issue of possible relaxation pathways for the excitation of the DPP chromophore. To do this, we performed both DFT and high-level calculations of the electronic structure of the DPP^Th^-NN_2_ diradical. In this paper, we described in detail the synthesis of DPP^Th^-NN_2_, single crystal analysis, insights into electronic structure, and the results on the continuous wave, echo-detected and time-resolved EPR study.

## Results and discussion

### Synthesis and structure of the DPP^Th^-NN_2_ diradical

**Scheme 1:**
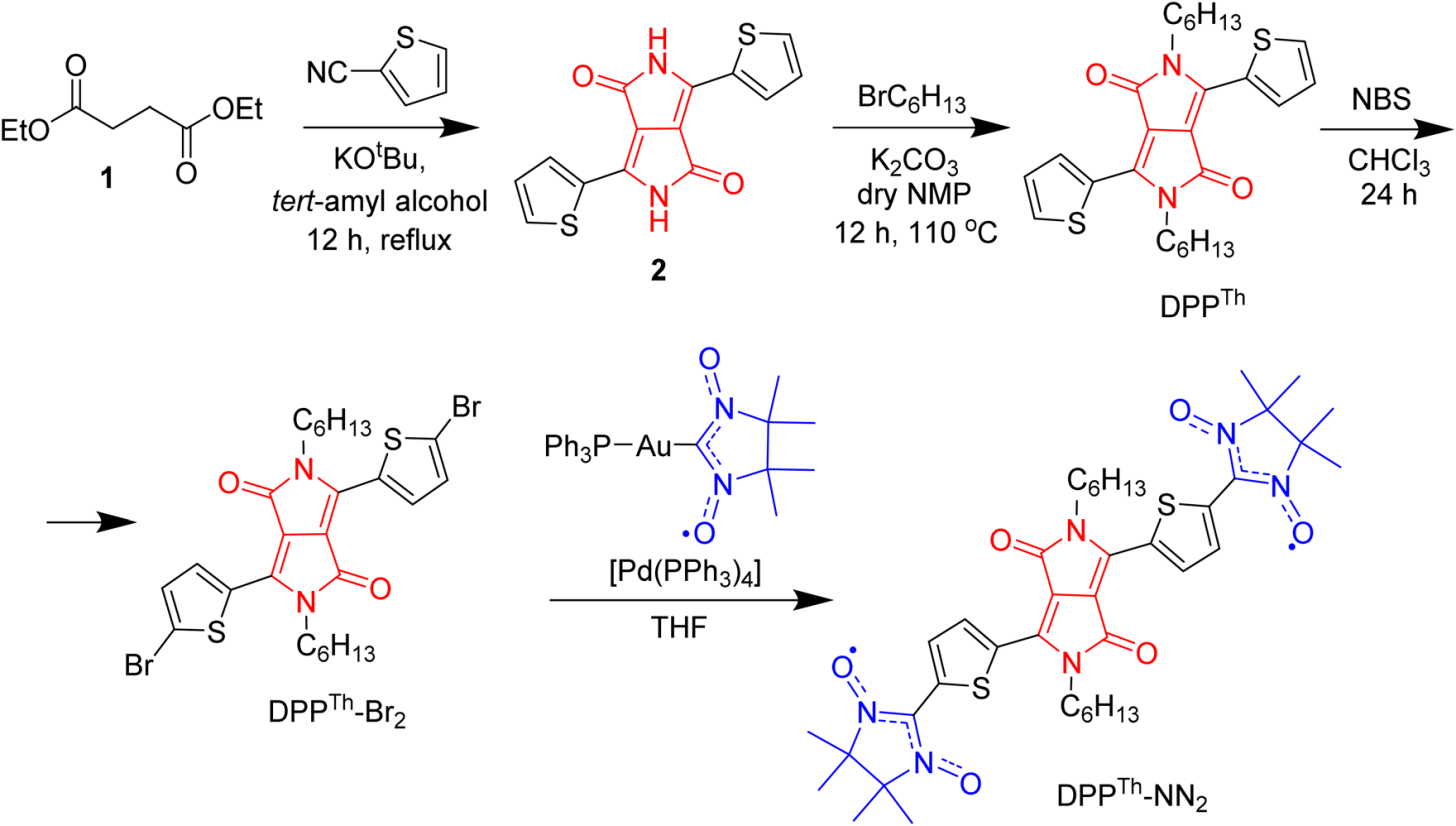
Synthesis of the DPP^Th^-NN_2_ nitronyl nitroxide diradical.

The synthetic route to the DPP^Th^-NN_2_ diradical is shown in Scheme 1. The diradical precursor, the corresponding dibromo-substituted DPP^Th^-Br_2_, was synthesized according to the literature-reported procedure^6^ with necessary modifications. To introduce two nitronyl nitroxide (NN) groups into the DPP^Th^ core, we chose a palladium catalysed cross-coupling of bromo-derivative of DDP with the (nitronyl nitroxide-2-ide)(triphenylphosphine)gold complex. This method, originally proposed by Keiji Okada,^7^ with the recent significant improvements^8,9^ has become an efficient protocol for the directed synthesis of nitronyl nitroxides.^10–12^ Thus, a new path has been opened to various in-demand nitronyl nitroxides for a variety of research fields, including radical chemistry, the creation of high-spin systems and rechargeable batteries, the design of molecule-based magnets and molecular units for spintronics.^13,14^ Naturally, using the described approach, the DPP^Th^-NN_2_ diradical was successfully obtained in a moderate yield of ∼72% when using Pd(PPh_3_)_4_ catalyst (Scheme 1).

In chemistry of diradicals, X-ray diffraction analysis is of paramount importance because it unveils valuable information about molecular organization and interactions in the solid state. We succeeded in growing single crystals of DPP^Th^-NN_2_ by slow diffusion of methanol into its solution in CH_2_Cl_2_ at 5 °C. The crystalline product appeared to be stable at ambient conditions and no phase transitions were observed in the DSC analysis in the temperature range from 20 °C to the melting point of 216 °C (see ESI†).

**Fig. 2:**
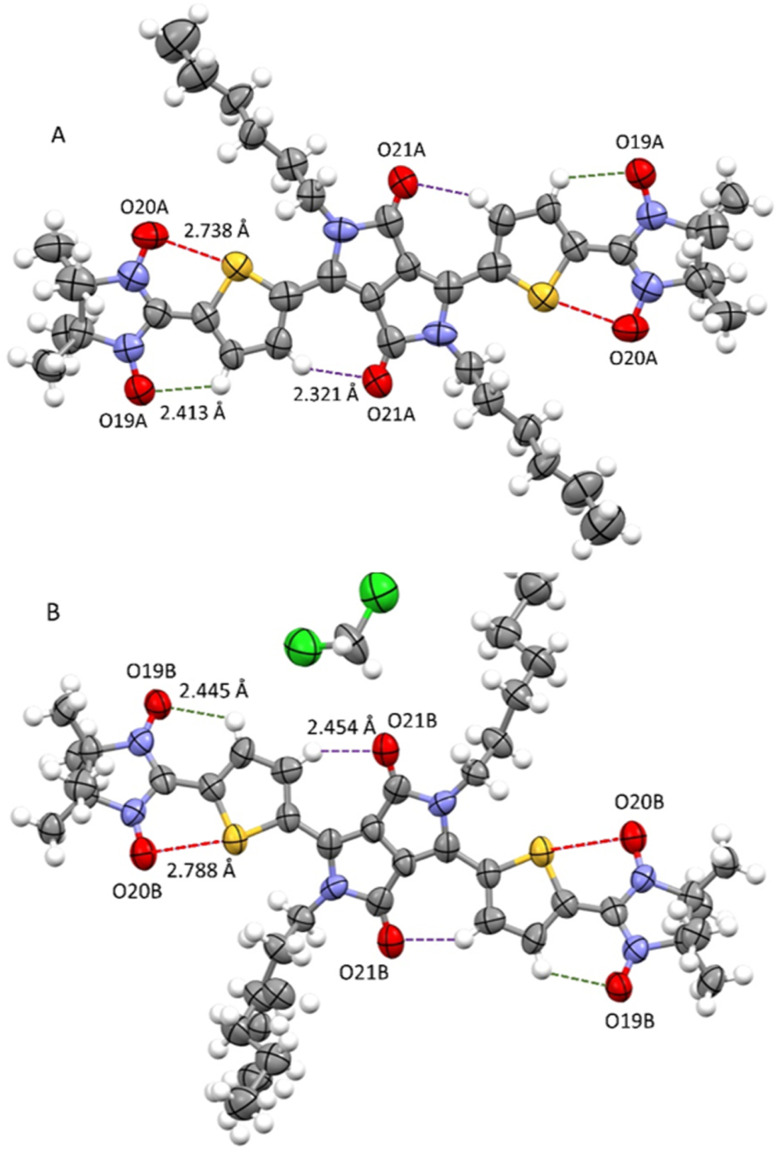
Structures of crystallographically independent molecules A and B (with solvate CH_2_Cl_2_) in crystals of the DPP^Th^-NN_2_ diradical.

At room temperature, attempts to perform an X-ray diffraction analysis of DPP^Th^-NN_2_ single-crystal samples failed. Cooling the single-crystal sample at 193 K led to the freezing of the alkyl-chain movement, which made it possible to solve the molecular and crystal structure of the DPP^Th^-NN_2_ diradical. According to XRD, the crystal structure of DPP^Th^-NN_2_ is centrosymmetric (space group *P*1̄) and contains two crystallographically independent molecules in an asymmetric unit (hereafter called as A and B). The molecular structures of A and B DPP^Th^-NN_2_ molecules are depicted in Fig. 2. Bond lengths and angles in both forms of the molecule are similar (Table S1†) and comparable to typical values for similar molecular fragments, as indicated by a search in the Cambridge Structural Database using the Mogul program.^15^

In the DPP^Th^-NN_2_ diradical, the π-conjugated system, consisting of the DPP core, thiophene rings, and nitronyl nitroxide moieties, presents high planarity with small interplanar twist angles. In molecules A and B, dihedral torsion angles between planes of the nitronyl nitroxide groups and planes of the nearest side thiophene rings are 2.90 and 13.24° respectively. In turn, the two thiophene rings are imposed in anti-orientation with respect to each other and twisted by 9.16° (in molecule A) and 20.69° (in molecule B) relative to the mean plane of DPP^Th^. The observed planarity of the thiophene–nitronyl nitroxide system leads to short intramolecular H-bonds O⋯H–C (2.41 and 2.45 Å, dotted lines in Fig. 2), as well as extremely short contacts between O_NO_ and S atoms (2.74 and 2.79 Å) compared to the sum of the van der Waals radii of the S and O atoms (3.32 Å, dashed purple lines in Fig. 2). Small torsion angles provide efficient conjugation along the backbone, thereby facilitating the noticeable transfer of spin-polarization from the NN radical units to the DPP^Th^ chromophore, as observed in the EPR spectra and demonstrated by DFT calculations (Fig. S6, ESI†).

**Fig. 3:**
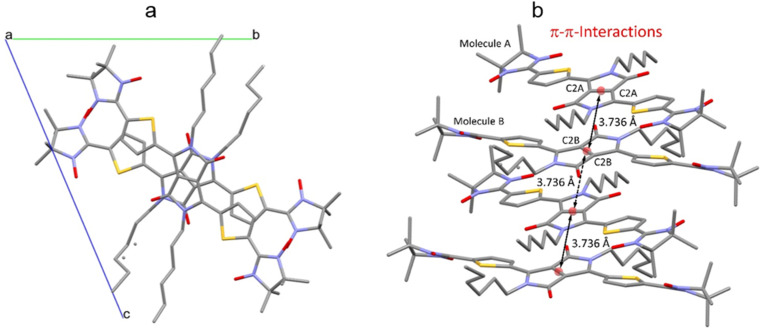
(a) Solid state molecular packing of DPP^Th^-NN_2_ with π–π-interactions; (b) side view of DPP^Th^-NN_2_ with π–π-distances between centroids of the DPP^Th^ moieties.

**Fig. 4:**
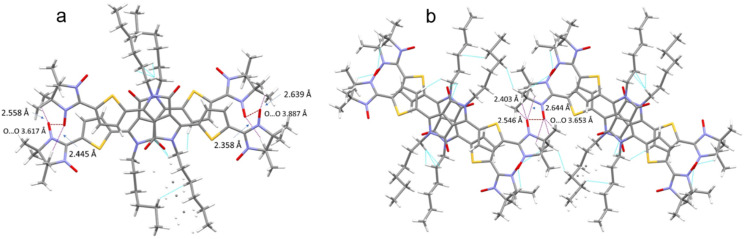
(a) Short C–H⋯O_NO_ contacts and the shortest O⋯O distances between neighbouring DPP^Th^-NN_2_ molecules in a stack and (b) between DPP^Th^-NN_2_ molecules belonging to different stacks.

The crystal packing shows that the molecules adopt weak intermolecular π–π stacking separated by distance of 3.74 Å between centroids of the DPP^Th^ moieties (Fig. 3). Inside such stacks, short intermolecular contacts C–H⋯O_NO_ (2.36, 2.45, 2.56, and 2.64 Å) are realized between the oxygen atoms of the nitroxide fragments and the hydrogen atoms of the methyl groups (Fig. 4a). Similar contacts are often observed in crystals of nitronyl nitroxides, and they are characterized by a rather high binding energy.^16^ Because of this, interactions of this type have a significant effect on the motif of the crystal packing of nitroxide radicals. The most important consequence of C–H⋯O_NO_ interactions is the geometry of the mutual arrangement of nitroxide groups and, above all, the distances between the oxygen atoms of paramagnetic centers, since the latter predetermine the magnetic properties of paramagnetic samples. Moreover, C–H⋯O_NO_ interactions predetermine the relative rotation angle of molecules in the stacks, as well as the shortest intermolecular distances between nitroxide oxygen atoms (3.617 and 3.887 Å). In addition, one can see multiple short intermolecular C–H⋯O_NO_ contacts (2.40, 2.55, and 2.64 Å) between the DPP^Th^-NN_2_ diradicals belonging to neighbouring stacks (Fig. 4b). These interactions lead to the shortest intermolecular distances between the nitroxide oxygen atoms, equal to 3.653 Å. On the whole, in the solid phase of DPP^Th^-NN_2_, the shortest O⋯O distances between O atoms of nitroxide groups exceed 3.617 Å, which is much greater than the sum of the van der Waals radii of O atoms (3.04 Å). Therefore, in the crystalline phase of DPP^Th^-NN_2_ diradicals, both intra- and intermolecular exchange interactions should be weak.

### DFT and *ab initio* calculations of the electronic structure and magnetic properties of the DPP^Th^-NN_2_ diradical

To better understand the magnetic and spectroscopic properties of the DPP^Th^-NN_2_ diradical, we performed a series of DFT and *ab initio* calculations. All calculations were performed for model geometries that differ from the geometry of the DPP^Th^-NN_2_ by replacing the long *n*-hexyl substituents with methyl groups. In addition, two types of model geometry were used in the calculations: one was based on the XRD analysis, and the other was optimized in a toluene solution.

#### Calculations of intra- and intermolecular exchange interactions

Since the DPP^Th^-NN_2_ diradical has SOMOs of a disjoint type (Fig. S7, S8, ESI and discussion ibid†), the parameters of the intramolecular exchange interaction between the NN fragments were calculated at the SA-CASSCF/NEVPT2 level of theory.^14^ The largest active space used in these calculations consisted of 14 electrons on 13 MOs (Fig. S8, ESI†). For the model geometry based on XRD analysis, the parameter *J* was calculated to be −11.3 cm^−1^ (for molecule A) at the highest level of calculations. As expected, the BS-DFT calculations overestimated absolute value of *J* (−60, −53 and −79 cm^−1^ with B3LYP, M06 and M06-2X functionals, respectively). Thus, the intramolecular exchange interaction in the diradical is indeed very weak and antiferromagnetic, and the ground state of the diradical is diamagnetic.

We also calculated the parameters of intermolecular exchange interactions of neighboring radical fragments in the stack. Interestingly, for a pair of diradicals, *J* parameters of different sign (−5.6 and 6.4 cm^−1^) were predicted for two pairs of adjacent radical fragments (Fig. S9, ESI†). An antiferromagnetic exchange interaction was predicted in the case when ONCNO fragments of neighboring radical fragments are almost parallel, and a ferromagnetic one, in the case of a significant deviation from parallel arrangement of the corresponding fragments.

#### Electronic absorption spectra of DPP^Th^ and DPP^Th^-NN_2_

**Fig. 5:**
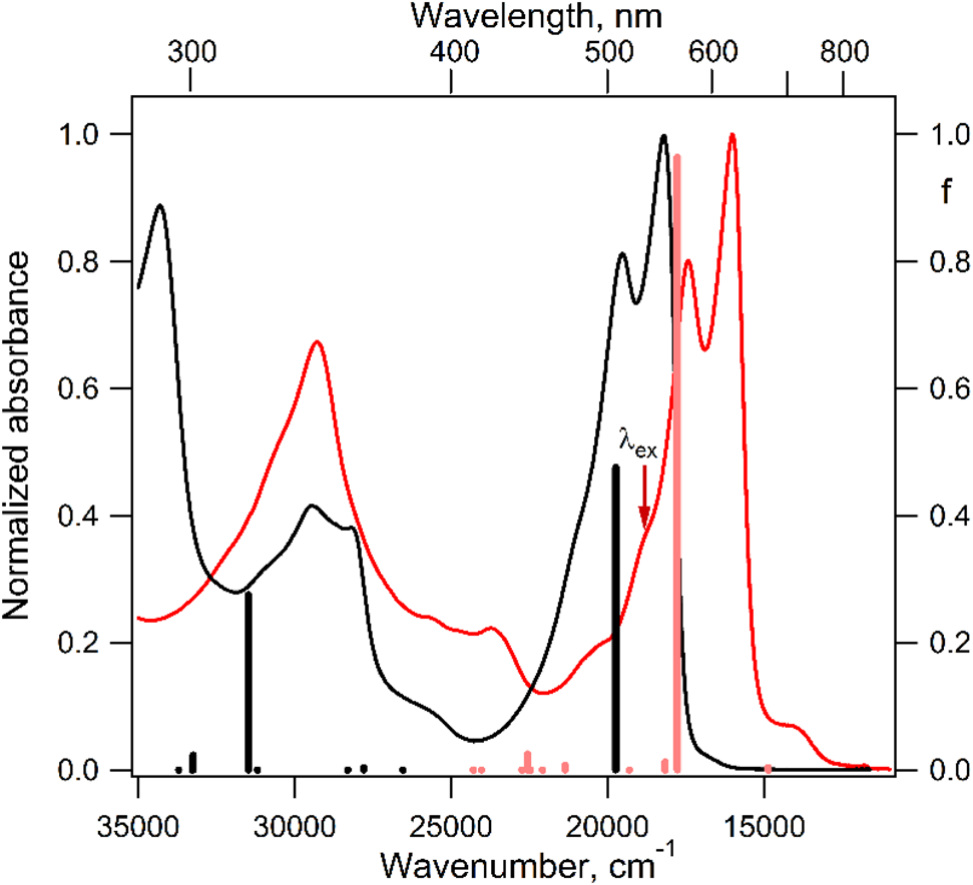
Normalized electronic absorption spectra of the DPP^Th^ precursor (black curve) and DPP^Th^-NN_2_ diradical (red curve) measured in toluene at room temperature, as well as the calculated positions and oscillator strengths (*f*, right axis) of electronic transitions depicted as black bars for DPP^Th^ and red bars for DPP^Th^-NN_2_.

**Fig. 6:**
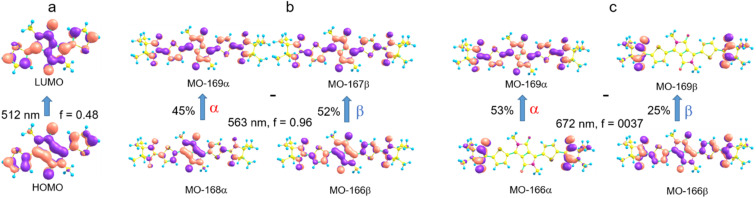
Molecular orbitals (MO) involved in the long wavelength transition in the DPP^Th^ spectrum (a), in the intense long wavelength transition in the DPP^Th^-NN_2_ spectrum (b), and in the weak transition at 672 nm (c).

Before proceeding to the calculation and analysis of exchange interactions in the excited states of the DPP^Th^-NN_2_ diradical, it is reasonable to interpret and analyze its electronic absorption spectrum and compare it with the spectrum of the parent DPP^Th^. Fig. 5 displays the electronic absorption spectra of both the DPP^Th^ and DPP^Th^-NN_2_ in toluene solution at room temperature. The DPP^Th^ shows an intense absorption band in the visible region with two maxima; this pattern is characteristic of diaryl-substituted DPP core.^2*f*,*g*^ According to the TD-B3LYP calculation, this structured band corresponds to a single electronic excitation (507 nm, *f* = 0.48, Fig. 5), in which the electron promotion from the highest occupied molecular orbital (HOMO) to the lowest unoccupied molecular orbital (LUMO) dominates (Fig. 6a). The next transition with a much smaller intensity is predicted at 360 nm (*f* = 0.0034). Thus, the maxima at 550 and 512 nm and the shoulder at about 480 nm represent the vibrational structure of the long wavelength band (with *ν* ∼ 1350 cm^−1^).

The electronic absorption spectrum of the DPP^Th^-NN_2_ diradical contains a very similar intense absorption band with the same structure as in the DPP^Th^ spectrum, but noticeably red-shifted. Note that the DPP^Th^-NN_2_ spectrum was calculated for the high-spin triplet state of the diradical. The wavefunction of the ground open-shell singlet cannot be correctly described in the one-determinant approximation, and thus its UV-Vis spectrum cannot be calculated at the TD-DFT level. Fig. S7 (ESI†) represents the MO diagram with a series of α- and β-type MOs involved in electronic excitations of the DPP^Th^-NN_2_ diradical. It can be seen that highest occupied and lowest unoccupied MOs of both α- and β-types are similar to the HOMO and LUMO of the DPP^Th^ (Fig. 6a) and differ from them only in some delocalization to NN fragments. According to the TD-UB3LYP calculation, the intense structured band in the spectrum of diradical also corresponds to a single intense transition (563 nm, *f* = 0.96, Fig. 5), which in turn corresponds to the HOMO → LUMO promotions for α- and β-electrons (Fig. 6b). Since these HOMO and LUMO are similar to those of the parent DPP^Th^ (except for a slight delocalization), the intense long wavelength bands of the precursor and diradical have very similar shape and intensity, but the diradical band is red-shifted.

In addition, the diradical spectrum shows a weak feature (around 680 nm) at the tail of the absorption band discussed above. According to the calculations, the DPP^Th^-NN_2_ has two electronic transitions in this region: at 672 nm (*f* = 0.0037) and 673 nm (*f* = ∼10^−8^). In turn, the MO diagram (Fig. S7, ESI†) shows that two SOMOs (MO-166α and MO-167α) localized on the NN fragments are practically degenerate and lie below the HOMO by about 0.2 eV. The corresponding unoccupied MO-168β and MO-169β localized on the NN fragments (partners of the α-SOMOs) are also practically degenerate and lie above LUMO by about 0.2 eV. It is these MOs, along with HOMO and LUMO, that are involved in long wavelength low intensity transitions. Both transitions consist mainly of two contributions: the promotion of an α-electron from the SOMO to the LUMO and the promotion of a β-electron from the HOMO to the SOMO partner (Fig. 6c). These transitions are more complex than those previously calculated for a number of nitronyl nitroxide radicals.^14^

#### Analysis of the spin-Hamiltonian parameters in the excited states

In the excited states of the DPP^Th^-NN_2_ diradical, a much stronger exchange interaction is expected. For example, the exchange interaction of the local excited triplet state of the DPP^Th^ core with the doublet states of the NN fragments should lead to four excited states: a singlet, two triplets, and a quintet. Knowledge of the sequence and splitting of these multiplets, as well as zero-field splitting of triplet and quintet states, is very important for the interpretation of time-resolved and echo-detected EPR spectra, and the phase relaxation kinetics in the presence and absence of laser radiation.

**Fig. 7:**
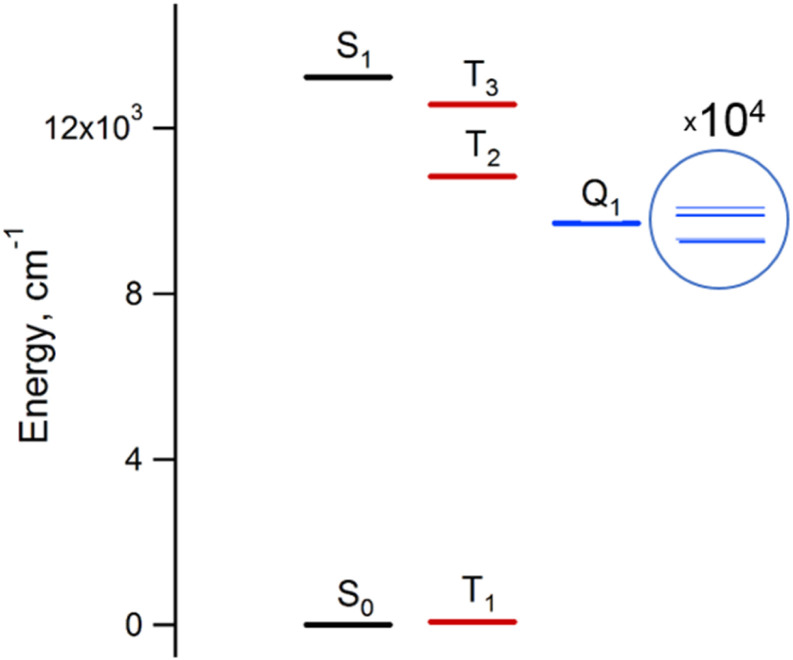
Energy diagram of the six lowest energy states of the DPP^Th^-NN_2_ diradical. The energies were calculated at the CASSCF(14,13)/NEVPT2/def2-TZVP level for the optimized geometry. The splitting of the lowest excited quintet state is calculated at the CAS(10,10)/QDPT level.

The results of the high-level calculations for the six low-energy DPP^Th^-NN_2_ multiplets are presented in Fig. 7 and S10 (ESI†), as well as in Table 1. Calculations were carried out both for the optimized and XRD-based geometries. An analysis of the CASSCF wave functions demonstrates that the Q_1_ state, as well as T2, T3, and S1 states, actually arose as a result of the exchange interaction of the local triplet state corresponding to the HOMO → LUMO excitation (Fig. 6a) with NN radical fragments (for details, see ESI, Section 3†). The energy splitting between these multiplets corresponds to the exchange interaction of the local triplet state with doublets of radical fragments (with parameter *J*_1_) and between radical fragments (with *J*_2_), respectively (Table 1). Results of Table 1 show that the exchange interaction of the radical fragments is weak and antiferromagnetic for both the ground and triplet excited states. In turn, the exchange interaction between the local triplet state and NN fragment is strong and ferromagnetic. The latter can be explained by the McConnell I mechanism,^17^ as carbon atom of ON-C-NO fragment has high negative spin population and is bound with carbon atom of thiophen ring with high positive spin population (Fig. S11†).

**Table 1:** Results of the CASSCF(14,13)/NEVPT2/def2-TZVP calculations of the low-energy spectrum of the DPP^Th^-NN_2_, consisting two singlet, three triplet and one quintet states, as well as the parameters of corresponding exchange interactions^a^ and the zero-field splitting (ZFS) parameters (*D*, *E*/*D*)

Electronic state	*E*, cm^−1^ and *J*, cm^−1^ (mT)		Optimized geometry	
	XRD geometry	Opt. geometry	*D*, cm^−1^ (in mT)	*E*/*D*
S_0_	0	0	No	No
T_1_	22.6	68.2	−6.5 × 10^−4^ (−0.70)^b^	0.077^b^
			−6.8 × 10^−4^ (−0.73)^c^	0.014^c^
*J*, cm^−1^	−11.3 (−1.21 × 10^3^)	−34.1 (−3.7 × 10^3^)	—	—
Q_1_	11 603	9698	−0.019 (−20.3)^b^	0.05^b^
T_2_	12 052	10 832	−0.108 (−116)^b^	0.01^b^
T_3_	12 570	12 011	0.052 (55.6)^b^	0.02^b^
S_1_	13 067	13 234	No	No
*J*_1_, cm^−1^	244	588	—	—
*J*_2_, cm^−1^	−10.2	−15.5	—	—
T_1_ of DPP^Th^	—	—	−0.042 (−44.9)^c^	0.20^c^

a Parameters *J*_1_ and *J*_2_ of the exchange interaction correspond to the spin Hamiltonian of the form 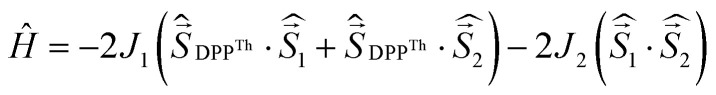, where 
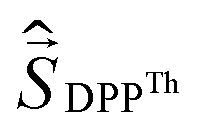
 is the spin of the DPP^Th^ core triplet state, and 
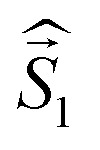
 and 
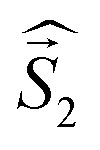
 correspond to the NN radical fragments.

b ZFS parameters estimated at the CASSCF(10,10)/QDPT level.

c ZFS parameters estimated at the RO-B3LYP level.

For the parent DPP^Th^, previous femtosecond transient absorption spectroscopy and time-correlated single-photon counting studies^18^ have demonstrated that the singlet excited S_1_ state of this compound relaxes directly to the ground state *via* internal conversion and fluorescence, bypassing the triplet state. For the DPP^Th^-NN_2_ the energy diagram is much more complicated. Excitation of the diradical by the 532 nm laser pulse leads to both singlet and triplet states arising from the exchange interactions of NN fragments through the excited singlet state localized mainly on the DPP^Th^ core. However, as was discussed previously, slightly below this pair of states, there are also states responsible for a weak feature in the UV-Vis spectrum (Fig. 5) (4 states in total - two triplets and two singlets). Finally, as shown by calculations, this sequence of excited states is closed by singlet, triplet, and quintet states presented in Fig. 7.

Thus, in the case of the DPP^Th^-NN_2_ diradical, between the state populated upon excitation and the ground state there are a large number of states, the energy gaps between which are small. In addition, among these states there are pairs of very close singlet and triplet levels that arise due to the exchange interaction of NN fragments. Therefore, one might expect a very fast conversion of the excited state (singlet or triplet), which occurs immediately after the excitation, into the Q_1_ state. At room temperature, the Q_1_ state is expected to be short-lived due to thermally activated relaxation through the T_2_ state. However, such a process is absent at cryogenic temperatures, and one can expect a sufficiently long-lived Q_1_ state at these temperatures.

#### The steady-state and echo-detected EPR spectra of the DPP^Th^-NN_2_

**Fig. 8:**
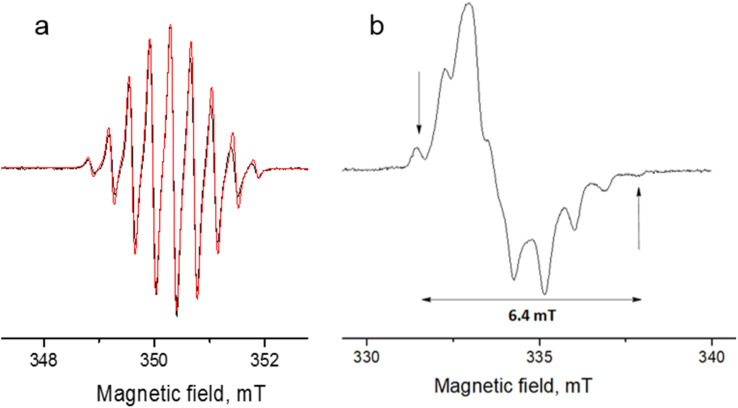
Experimental (black) and simulated (red) continuous-wave (CW) X-band EPR spectra of DPP^Th^-NN_2_ in toluene at room temperature (a) and in frozen toluene at 135 K (b) (*C* ≈ 0.1 mM).

Although the ground state of the DPP^Th^-NN_2_ diradical is singlet and diamagnetic, its triplet state at room temperature is substantially populated (about 70% using predicted *J* value). Thus, the EPR spectrum of DPP^Th^-NN_2_ in a toluene solution at ambient temperature (Fig. 8a) was recorded, and it is characteristic of bis(nitronyl-nitroxide) systems with an intramolecular exchange interaction significantly exceeding the hyperfine coupling (|*J*| ≫ *A*_N_), which is also consistent with the results of our calculations (|*J*| = 3.7 × 10^3^ mT, Table 1). The spectrum has a center at *g*_iso_ = 2.0065 and contains nine lines due to the coupling of two unpaired electrons with four equivalent ^14^N nuclei with |*A*_N_|/2 ∼ 0.374 mT, which is experimental evidence of the diradical nature of DPP^Th^-NN_2_.

The EPR spectrum of the DPP^Th^-NN_2_ diradical in a frozen toluene solution at 135 K (Fig. 8b) is difficult to interpret, since the symmetry is partly lost and some anisotropic components may overlap in |Δ*m*_S_| = 1 region. This results in a different number of shoulders in the outermost regions of the spectrum. Therefore, the zero-field splitting parameters can be estimated roughly from the low temperature EPR experiment. However, clear evidence of a diradical character comes from the observed |Δ*m*_S_| = 2 transition. This signal has a low signal-to-noise ratio (S/N), because the average value of the N⋯N, O⋯O and N⋯O distances is large and estimated at 15.3 Å (for both the XRD and optimized structures), which implies a small |*D*| and, accordingly, a low probability of this transition. In the point dipole approximation, a distance of 15.3 Å leads to |*D*| = 0.81 mT (7.6 × 10^−4^ cm^−1^). Note that this value cannot explain the rather large width of the EPR spectrum (Fig. 8b). However, the calculation of the spin–spin contribution at the DFT level gave an even smaller but close value: *D* = −0.73 mT (−6.8 × 10^−4^ cm^−1^) and *E*/*D* = 0.014.

**Fig. 9:**
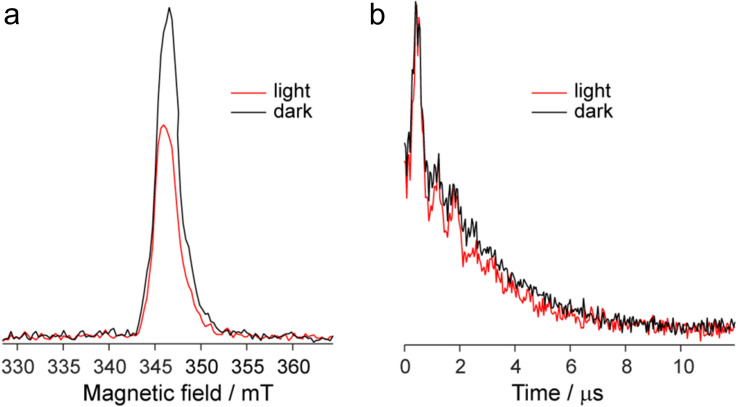
Echo-detected EPR spectra (a) and phase relaxation kinetics (two-pulse echo decay *vs.* interpulse delay) (b) the DPP^Th^-NN_2_ diradical in toluene glass at *T* = 10 K in the presence/absence of photoexcitation. Kinetic curves are normalized to the maximum.

Fig. 9a shows the echo-detected (ED) EPR spectra of the DPP^Th^-NN_2_ diradical recorded at 10 K under the same conditions in the presence and absence of pulse laser excitation. The spectrum recorded in the absence of irradiation features one slightly asymmetric EPR line, the maximum of which corresponds to *g* = 2.008. This signal apparently corresponds to the lowest triplet state of the DPP^Th^-NN_2_ diradical (T_1_), and, according to calculations, its population at 10 K is low, although it cannot be ruled out that *E*(T_1_) may be substantially overestimated.

In the presence of laser radiation, the observed EPR signal retains its shape, but becomes less intense. Based on the quantum chemical calculations and discussion presented above, one should expect that the lowest excited quintet state (Q_1_) may be populated upon excitation, thus being paramagnetic. However, still we do not observe any reliable spectral manifestations of such state. In addition, the transverse relaxation curves in the presence and absence of photoexcitation are remarkably similar (Fig. 9b), indicating that they refer to the same paramagnetic species.

We also applied time-resolved (TR) EPR technique to probe the intermediate species formed upon photoexcitation of DPP^Th^-NN_2_ in toluene glass at 80 K. TR EPR is based on continuous wave detection and typically is more sensitive to photoexcited paramagnetic species compared to pulse EPR. However, no TR EPR signals were observed. In addition, the sample with pure DPP^Th^ moiety (without radicals attached) has been investigated with the same zero TR EPR signal. The failure to detect the TR EPR signal in the case of DPP^Th^ is consistent with the relaxation of the S_1_ state directly to the ground state, as discussed above. The failure in the case of the diradical may be due to the short lifetimes of T_2_, T_3_ and Q_1_ states, if they are actually formed.

The observed decrease of the echo signal under light can be assigned to a slight heating of the sample. Indeed, an insignificant heating by a few degrees (3–5 K) can noticeably modify EPR signal at 10 K, which is proportional to 1/*T* (see, *e.g.* ref. 19 and 20). At the same time, the opposite effect of heating should be present due to the higher population of T_1_ state at higher temperatures. Apparently, the former mechanism dominates, causing spectral changes shown in Fig. 9a. Additional perturbation of resonator under laser radiation can also contribute to the observed signal decrease.

## Conclusions

In summary, we have synthesized a DPP-derivative bearing two radical groups, *i.e.* bis(thiophen-2-yl)-2,5-dihydropyrrolo[3,4-*c*]pyrrole-1,4-dione terminally capped with nitronyl nitroxide groups (DPP^Th^-NN_2_). Notably, we used palladium-catalyzed cross-coupling reaction of the corresponding dibromo-derivative with the (nitronyl nitroxide-2-ide)(triphenylphosphine)gold complex as a convergent step to synthesize DPP^Th^-NN_2_ diradical. The incorporation of two radicals resulted in interesting spectroscopic, photophysical, and magnetic properties, and the expected unusual excited-state dynamics. A computational study predicted that the DPP^Th^-NN_2_ diradical has singlet ground state with a small singlet–triplet energy gap Δ*E*_ST_ of about 0.2 kcal mol^−1^ in solution. This value is consistent with strong on the EPR scale exchange interactions observed in the EPR. The inclusion of two NN radicals leads to an increase in the number of excited states both due to excitations involving orbitals localized on NN fragments and due to the exchange interaction of these fragments. Consequently, the density of states increases and, in turn, the energy gaps between them decrease. This effect, combined with the removal of spin prohibition for certain transitions, is expected to significantly speed up the relaxation process of the excitation initially localized on the DPP^Th^ core. Although we were unable to experimentally detect the low-energy quintet state, likely due to its brief lifetime or low yield, future studies using ultrafast techniques, such as femtosecond spectroscopy, could potentially reveal its properties.

## Experimental and computational details

### Materials and instrumentation

All chemicals and reagents were purchased from commercial suppliers and used without further purification. Solvents used for spectroscopic measurements were spectral grade quality. 2,5-Dihydro-1,4-dioxo-3,6-dithienylpyrrolo[3,4-*c*]-pyrrole (2) was synthesized by adapting reported procedure.^21^ All reactions were monitored by thin-layer chromatography (TLC) carried out on silica gel plates. Preparative separations were performed by column chromatography on silica gel grade 60 (0.040–0.063 mm) from Merck.

^1^H and ^13^C NMR spectra were recorded on Bruker Avance 300 (300 MHz) spectrometer. The chemical shifts were reported in ppm and referenced to the residual solvent peak. s = singlet, d = doublet, t = triplet, m = multiplet, b = broad. Infrared spectroscopy measurements were conceded on Nicolet 730 FTIR spectrometer equipped with an attenuated total reflection (ATR) setup. The UV/Vis spectra were recorded at 298 K with a PerkinElmer Lambda 900 spectrophotometer. Matrix-assisted laser desorption ionization time-of-flight (MALDI-TOF) mass spectra were acquired with a Bruker Reflex II MALDI-TOF mass spectrometer, calibrated against a mixture of C_60_/C_70_. The X-ray crystallographic data for the molecules were collected on a STOE IPDS 2T diffractometer using Cu-Kα IμS source. Crystal structure contains two independent molecules of NN_2_-DPP (A and B) both of them are located on an centre of inversion which result in a *C*_i_ symmetry. The alkyl chains are disordered. One solvent molecule (CH_2_Cl_2_) complete the unit cell.

### Computational details

All quantum chemical calculations were performed for model structures that differ from the structures of the compounds under study by replacing the long *n*-butyl substituents with methyl groups. This substitution should only slightly affect the electronic and spectral properties of the parent DPP^Th^ and the DPP^Th^-NN_2_ diradical.

To analyze magnetic properties of polycrystalline samples, all quantum chemical calculations were performed for the model geometry of DPP^Th^-NN_2_ diradicals and their pairs obtained from XRD analysis. The parameters of the intramolecular exchange interaction 
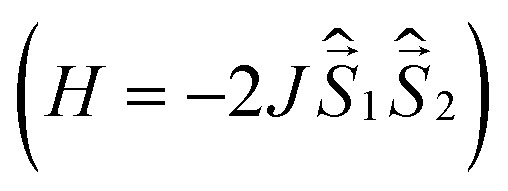
 were computed using the accurate *ab initio* CASSCF^22^ and CASSCF/NEVPT2 (ref. 23 and 24) procedures. To calculate the *J* parameters for intermolecular exchange interactions, the spin-unrestricted broken-symmetry (BS) approach^25^ at the BS-B3LYP/def2-TZVP level of theory^26–28^ using the Yamaguchi formula^29^ was utilized
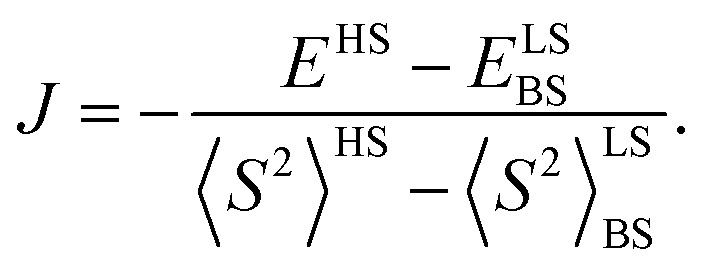


To rationalize the results of experiments performed in solutions, calculations were carried out using the model geometry optimized in toluene solution at the UB3LYP/def2-TZVP and UM06-2X/def2-TZVP levels of theory;^30^ the solvent was taken into account according to the CPCM model.^31^

The energies of electronic transitions and their oscillator strengths in the electronic absorption spectra of DPP^Th^ and DPP^Th^-NN_2_ were calculated using the time-dependent DFT^32^ at the TD-B3LYP/def2-TZVP level. In the case of DPP^Th^-NN_2_, the calculations were performed for the high-spin triplet state of diradical. The zero-field splitting parameters (ZFS, *D* and *E*/*D*) for the lowest-energy triplet state of the DPP^Th^-NN_2_ diradical was calculated at the RO-BP86/def2-TZVP level,^33,34^ while the *g*-tensor and hyperfine splitting tensors at the B1LYP/def2-TZVP level.^35^

The energies of low-lying states (2 singlets, 3 triplets and 1 quintet) were also calculated at the CASSCF(14,13)/NEVPT2/def2-TZVP level. Moreover, the splittings of the triplet and quintet multiplets (ZFS) due to the dipolar spin–spin interaction and spin–orbit coupling (SOC) were evaluated using CASSCF(10,10) wavefunctions and quasi-degenerate perturbation theory (QDPT),^36^ as realized in the CASSCF and MRCI modules of the ORCA 5.0.5 software package.^37^ The contribution of SOC was calculated using the mean-field formalism (SOMF(1X))^38^ and found to be negligible. To the best of our knowledge, this work is the first to provide high-level calculations of the low-energy spectrum of a dye substituted with two radical fragments.

The same software package was used for all other calculations. The molecular orbitals were visualized using the Chemcraft software.^39^

### Synthetic methods and characterization

#### 3,6-Di(thiophen-2-yl)-2,5-dihydropyrrolo[3,4-*c*]pyrrole-1,4-dione (2)^21^

To a 250 mL two-neck round bottom flask equipped with magnetic stirrer, potassium *t*-butoxide (6.5 g, 57.9 mmol), 2-thiophenecarbonitrile (1) (5.0 g, 45.9 mmol) and *tert*-amyl alcohol (30 mL) were added and the mixture was heated to 100 °C under a nitrogen atmosphere. At this temperature, a solution of di-ethyl succinate (3.85 g, 22.1 mmol) and *tert*-amyl alcohol (5 mL) was added to the reaction mixture over 1 h using a dropping funnel. The reaction mixture was stirred at 100 °C for 20 h, and then cooled to room temperature, neutralized with glacial acetic acid (35 mL), and gently reflux temperature for 1 h. The resulting pigment suspension was suspended in water–methanol mixture (1 : 1, 50 mL) and filtered to get pigment cake, which was washed with water–methanol mixture until no color found in washings. The crude compound was dried at 100 °C *in vacuo*, and obtained 4.0 g of compound 2. The product was used in the next step without further purification.

#### 2,5-Dihexyl-3,6-di(thiophen-2-yl)-2,5-dihydropyrrolo[3,4-*c*]pyrrole-1,4-dione (DPP^Th^)^40,41^

To a 250 mL two-neck round bottom flask equipped with magnetic stirrer, compound 2 (1.00 g, 3.33 mmol) and anhydrous K_2_CO_3_ (1.4 g, 10.1 mmol) were added in 100 mL of anhydrous *N*,*N*-dimethylformamide (DMF). The reaction mixture was heated to 120 °C under nitrogen for 1 h. Alkyl bromide (8.0 mmol) was then added dropwise, and the reaction mixture was further stirred and heated 12 h at 130 °C. The reaction mixture was cooled to room temperature; then it was poured into 100 mL of water, and the resulting suspension was stirred at room temperature for 1 h. The solid was collected by vacuum filtration, washed with copious amount of water, followed by methanol, and then air-dried. The crude product was checked on TLC and ^1^H NMR to confirm the isomer presence and it is found to be around 45 : 55 ratio based on ^1^H NMR spectra. The crude mixture was purified on chromatography using 3% ethyl acetate in hexane as an eluent, and the solvent was removed under vacuum to obtain a pure product DPP^Th^ in 40% yield. ^1^H NMR (300 MHz, CDCl_3_, ppm) *δ* = 8.92 (d, 2H Th–H), 7.64 (d, 2H, Th–H), 7.27 (m, 2H, Th–H), 4.07 (d, 4H, N–CH_2_), 1.74 (m, 4H, CH_2_–CH_2_), 1.41–1.28 (m, 12H), 0.87 (t, 12H). ^13^C NMR (125 MHz, CDCl_3_, ppm) *δ* = 161.37, 140.02, 135.21, 130.63, 129.77, 128.58, 107.70, 42.21, 31.39, 29.89, 26.53, 22.53, 13.98. MALDI-TOF, *M*_w_ 468.9518. Anal. calcd for C_26_H_32_N_2_O_2_S_2_: C, 66.63; H, 6.88; N, 5.98; S, 13.68. Found: C, 66.83; H, 7.01; N, 6.16; S, 13.42.

#### 3,6-Bis(5-bromothiophen-2-yl)-2,5-dihexyl-2,5-dihydropyrrolo[3,4-*c*]pyrrole-1,4-dione (DPP^Th^-Br_2_)

Compound DPP^Th^-Br was synthesized by following a modified procedure reported.^41^ To a 100 mL two-neck round bottom flask, compound DPP^Th^ (0.60 g, 1.28 mmol) was added and dissolved in to dry DMF (10 mL) under nitrogen atmosphere. The reaction mixture was cooled down to 0 °C in ice bath and *N*-bromosuccinimide (NBS) (0.46 g, 2.6 mmol) was added portion wise. It was stirred at room temperature for 12 h. This mixture was poured into water (100 mL) and stirred for 1 h. Solid was vacuum filtered and washed with MeOH and H_2_O to remove excess NBS. The crude solid was purified through column chromatography using hexane : DCM (1 : 1) as an eluent. Compound DPP^Th^-Br was obtained as a dark purple solid (0.58 g, yield 83%). ^1^H NMR (300 MHz, CD_2_Cl_2_, ppm) *δ* = 8.67 (d, 2H Th–H), 7.27 (d, 2H, Th–H), 3.98 (d, 4H, N–CH_2_), 1.64–1.74 (m, 4H), 1.30-8-1.40 (m, 12H), 0.89 (t, 6H). ^13^C NMR (75 MHz, CDCl_3_, ppm) *δ* = 160.95, 139.01, 135.32, 131.62, 131.12, 119.10, 107.90, 42.30, 31.90, 29.95, 26.80, 22.63, 14.07. UV/Vis (toluene): *λ*_max_: 550 nm. MALDI-TOF, Mw 626.47 (626.55). Anal. calcd for C2_6_H_30_Br_2_N_2_O_2_S_2_: C, 49.85; H, 4.83; Br, 25.51; N, 4.47; O, 5.11; S, 10.24. Found: C, 49.76; H, 4.75; Br, 26.05; N, 4.55; S, 9.89.

#### 3,6-Bis(5-(4,4,5,5-tetramethyl-4,5-dihydro-3-oxid-1-oxyl-1*H*-imidazole-2-yl)thiophene-2yl)-2,5-dihexyl-2,5-dihydropyrrolo[3,4-*c*]pyrrole-1,4-dione (DPP^Th^-NN_2_)

A solution of dibromide Br_2_-DPP (100 mg, 0.16 mmol), nitronyl nitroxide gold complex (218 mg, 0.35 mmol), and [Pd(PPh_3_)_4_] (20 mg) in THF (15 mL) was stirred at 65 °C in an argon atmosphere for 16 h. The reaction mixture was cooled to room temperature and solvent was evaporated. The residue was purified by column chromatography on silica gel by using CH_2_Cl_2_/ethyl acetate (10 : 1) solvent mixture as an eluent. The resulted product was recrystallized two times from a mixture of CH_2_Cl_2_ with MeOH to obtain diradical NN_2_-DPP (90 mg, 72 %). Compound DPP^Th^-NN_2_ was eluted with *R*_f_ = 0.7 (10 : 1 mixture of CH_2_Cl_2_/ethyl acetate); IR: *ν* = 2956, 2927, 2856, 1662, 1552, 1436, 1400, 1367, 1367, 1211, 1176, 1134, 1095, 870, 827, 729, 620, 540 cm^−1^; UV/Vis (toluene): *λ*_max_: two maxima at 573 and 624 nm and a shoulder at ∼680 nm; MALDI-TOF MS: *m*/*z* calcd. (%) for C_52_H_76_N_4_O_4_S_4_: 778.35 (100) [M]^+^, 779.36 (43) [M + 1]^+^; found: 778 (average *M*_W_); elemental analysis calcd: C 61.67, H 6.99, N 10.79, S 8.23; found: C 61.80, H 7.1, N 10.3, S 8.05. Melting point, 216 °C.

### Single-crystal XRD analysis

Single crystal X–ray diffraction data for DPP^Th^-NN_2_ were collected at 193 K on a STOE IPDS 2T diffractometer with Cu-K_α_ IμS mirror system (Table 2). The structure was solved using direct methods, expanded with Fourier techniques and refined with the SHELXT software package. All non-hydrogen atoms were refined anisotropically. Hydrogen atoms were included in the structure factor calculation on geometrically idealized positions. Crystallographic data have been deposited with the Cambridge Crystallographic Data Centre as supplementary publication no. CCDC 1908216.†

**Table 2:** Crystal data and details of experiment for DPP^Th^-NN_2_ diradical

Formula	C_40_H_54_N_6_O_6_S_2_, CH_2_Cl_2_
Molecular weight	863.93 g mol^−1^
Absorption	*μ* = 2.66 mm^−1^ correction with six crystal faces
Transmission	*T*_min_ = 0.7125, *T*_max_ = 0.9602
Crystal size	0.020 × 0.020 × 0.180 mm^3^, black needle
Space group	*P*1̄ (triclinic)
Lattice parameters (calculate from 11 587 reflections with 3.0° < *θ* < 62.2°)	*a* = 7.4729(10) Å, *α* = 67.095(10)°
	*b* = 16.121(2) Å, *β* = 88.242(11)°
	*c* = 19.743(3) Å, *γ* = 84.135(11)°
	*V* = 2179.4(5) Å^3^, *z* = 2, *F*(000) = 916.0
Temperature	193 K
Density	*d*_X-ray_ = 1.317 g cm^−3^

**Data collection**	
Diffractometer	STOE IPDS 2T
Radiation	Cu-K_α_ IμS mirror system
Scan – type	ω scans
Scan – width	1°
Scan range	2° ≤ *θ* < 68°
	−8 ≤ *h* ≤ 8, −19 ≤ *k* ≤ 18, −20 ≤ *l* ≤ 22
*Number of reflections*	
Measured	24 811
Unique	7446 (*R*_int_ = 0.183)
Observed	1621 (|*F*|/*σ*(*F*) > 4.0)

**Data correction, structure solution and refinement**	
Corrections	Lorentz and polarisation correction
Structure solution	Program: SHELXT-2014
Refinement	Program: SHELXL-2014 (full matrix). 550 refined parameters, weighting scheme
	*w* = 1/[*σ*^2^(*F*_o_^2^) + (0.2 × *P*)^2^] with (max(*F*_o_^2^,0) + 2 × *F*_c_^2^)/3. H-atoms at calculated positions and refined with isotropic displacement parameters, non H-atoms refined anisotropically
*R*-values	w*R*_2_ = 0.4395 (*R*_1_ = 0.1407 for observed reflections, 0.3430 for all reflections)
Goodness of fit	*S* = 0.881
Maximum deviation of parameters	0.001 × e.s.d
Maximum peak height in diff. Fourier synthesis	0.52, −0.92 e Å^−3^

## Photoinduced EPR experiments

Pulse EPR experiments were conducted at Bruker Elexsys E580 spectrometer at the Center of Collective Use ‘‘Mass spectrometric investigations’’ SB RAS at X-band (9 GHz). Spectrometer is equipped with flow helium cryostat (Oxford Instruments) and thermocontroller Lakeshore and allows measurements at *T* = 4–300 K. In order to measure echo-detected (ED) EPR spectra and transverse (phase) relaxation times (*T*_2_) we used standard two-pulse sequence with the pulse lengths being 10 and 20 ns for π/2 and π pulses, respectively. In case of *T*_2_ measurements, interpulse delay was incremented. Time-Resolved (TR) EPR experiments were performed using homemade spectrometer based on commercial Bruker EMX microwave bridge.

In all cases the studied compounds were dissolved in toluene in concentrations *ca.* 0.5 mM and placed into quartz sample tubes with outer diameter of 2.8 mm. The samples were degassed by a few freeze–pump–thaw cycles and then sealed off *in vacuo*. EPR studies were performed for two samples: DPP^Th^-NN_2_ biradical and its photosensitive moiety DPP^Th^ free of radical fragments.

Laser irradiation was provided by Nd:YaG LOTIS-TII system at 532 nm, power ∼ 20 mJ per pulse, 10 Hz repetition rate. In case of pulse EPR detection laser was synchronized with EPR spectrometer; laser pulse proceeded the first microwave pulse by 700 ns. In TR EPR experiments the detection was also synchronous with laser pulsing; microwave absorption was detected starting from 100 ns till 9 μs after the laser pulse.

## Conflicts of interest

There are no conflicts to declare.

## Supplementary Material

RA-014-D4RA00916A-s001

RA-014-D4RA00916A-s002
