# Supplementary material for: Synthesis and photoinduced behavior of DPP-anchored nitronyl nitroxides: a multifaceted approach
Source: RSC Adv. 2024 Feb 19;14(9):6178–89. doi: 10.1039/d4ra00916a (PMC10875417; doi:10.1039/d4ra00916a)
Supplement: RA-014-D4RA00916A-s001 [file RA-014-D4RA00916A-s001.pdf]

Electronic Supplementary Material (ESI) for RSC Advances.
This journal is © The Royal Society of Chemistry 2024

Synthesis and Photoinduced Behavior of DPP-Anchored Nitronyl Nitroxides: A Multifaceted Approach

Evgeny Tretyakov,[a]* Dmitry Gorbunov,[b] Nina Gritsan,[b]* Ashok Keerthi,[c] Martin Baumgarten,[d] Dieter Schollmeyer,[e] Mikhail Ivanov,[f] Anna Sergeeva,[a] Matvey Fedin[f]

[a] *N.D. Zelinsky Institute of Organic Chemistry, Russian Academy of Sciences, Leninsky Ave. 47, Moscow 119991, Russian Federation.*

[b] *V. V. Voevodsky Institute of Chemical Kinetics and Combustion, 3 Institutskaya Str., Novosibirsk 630090, Russian Federation.*

[c] *Department of Chemistry, School of Natural Sciences, the University of Manchester, Oxford Road, M13 9PL, UK.*

[d] *Max Planck Institute for Polymer Research, Ackermannweg 10, Mainz D-55128, Germany.*

[e] *Johannes Gutenberg-University Mainz, Duesbergweg 10-14, 55128 Mainz, Germany.*

[f] *International Tomography Center. 3a Institutskaya Str., Novosibirsk 630090, Russian Federation.*

Table of Contents:

1. Experimental Methods 2

2. Photo induced EPR-experiments 9

3. Electronic structure of the DPPTh-NN$_2$ diradical 10

1. Experimental Methods

1.1. Materials and Instrumentation

All chemicals and reagents were purchased from commercial suppliers and used without further purification. Solvents used for spectroscopic measurements were spectral grade quality. 2,5-Dihydro-1,4-dioxo-3,6-dithienylpyrrolo[3,4-*c*]-pyrrole (**2**) was synthesized by adapting reported procedure.[1] All reactions were monitored by thin-layer chromatography (TLC) carried out on silica gel plates. Preparative separations were performed by column chromatography on silica gel grade 60 (0.040 – 0.063 mm) from Merck.

^{1}H and ^{13}C NMR spectra were recorded on Bruker Avance 300 (300 MHz) spectrometer. The chemical shifts were reported in ppm and referenced to the residual solvent peak. s = singlet, d = doublet, t = triplet, m = multiplet, b = broad. Infrared spectroscopy measurements were conceded on Nicolet 730 FTIR spectrometer equipped with an attenuated total reflection (ATR) setup. The UV/Vis spectra were recorded at 298 K with a Perking-Elmer Lambda 900 spectrophotometer. Matrix-assisted laser desorption ionization time-of-flight (MALDI-TOF) mass spectra were acquired with a Bruker Reflex II MALDI-TOF mass spectrometer, calibrated against a mixture of C_{60}/C_{70}. The X-ray crystallographic data for the molecules were collected on a STOE IPDS 2T diffractometer using Cu-Kα IµS source. Crystal structure contains two independent molecules of DPP^{Th}-NN_2 (A and B) both of them are located on a centre of inversion which result in a C_i symmetry. The alkyl chains are disordered. One solvent molecule (CH_2Cl_2) completes the unit cell.

Cyclic voltammetry (CV) measurements were carried out with a computer-controlled GSTAT12 within a three-electrode cell in a CH_2Cl_2 solution of Bu_4NPF_6 (0.1 m) at a scan rate of 100 mV s^{-1} at room temperature. The electrochemical cell consists of three electrode systems with platinum disc as working electrode, platinum wire as counter electrode and an Ag electrode as a reference electrode. The potentials were calibrated using ferrocene as internal standard.

1.2. Synthetic Methods and Characterization

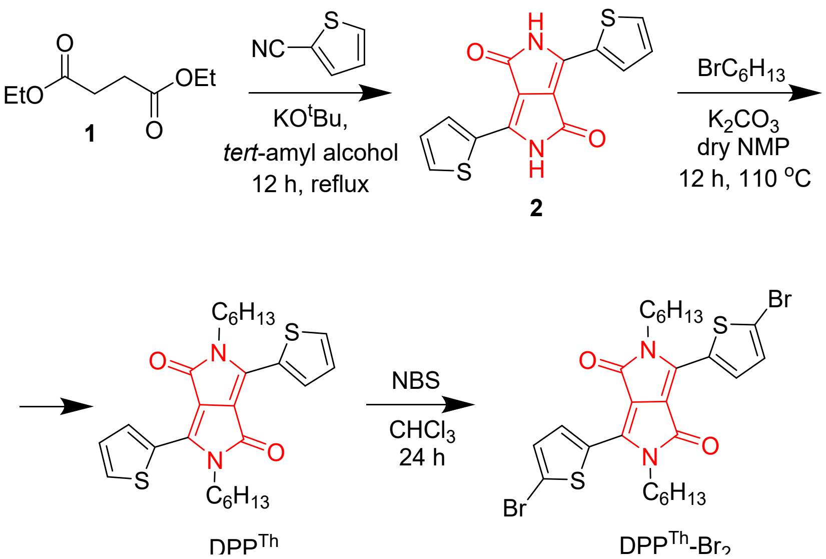

Scheme S1. Synthesis of DPPTh-Br$_2$ derivative.

3,6-Di(thiophen-2-yl)-2,5-dihydropyrrolo[3,4-c]pyrrole-1,4-dione (2).[1] To a 250 mL two-neck round bottom flask equipped with magnetic stirrer, potassium *t*-butoxide (6.5 g, 57.9 mmol), 2-thiophenecarbonitrile (5.0 g, 45.9 mmol) and *tert*-amyl alcohol (30 mL) were added and the mixture was heated to 100 °C under a nitrogen atmosphere. At this temperature, a solution of di-ethyl succinate (3.85 g, 22.1 mmol) and *tert*-amyl alcohol (5 mL) was added to the reaction mixture over 1 h using a dropping funnel. The reaction mixture was stirred at 100 °C for 20 h, and then cooled to room temperature, neutralized with glacial acetic acid (35 mL), and gently reflux temperature for 1 h. The resulting pigment suspension was suspended in water-methanol mixture (1:1, 50 mL) and filtered to get pigment cake, which was washed with water-methanol mixture until no colour found in washings. The crude compound was dried at 100 °C in vacuo, and obtained 4.0 g of compound **2**. The product was used in the next step without further purification.

2,5-Dihexyl-3,6-di(thiophen-2-yl)-2,5-dihydropyrrolo[3,4-c]pyrrole-1,4-dione (DPPTh).[2,3] To a 250 mL two-neck round bottom flask equipped with magnetic stirrer, compound **2** (1.00 g, 3.33 mmol) and anhydrous K_2CO_3 (1.4 g, 10.1 mmol) were added in 100 mL of anhydrous *N,N*-dimethylformamide (DMF). The reaction mixture was heated to 120 °C under nitrogen for 1 h. Alkyl bromide (8.0 mmol) was then added dropwise, and the reaction mixture was further stirred and heated 12 h at 130 °C. The reaction mixture was cooled to room temperature; then it was poured into 100 mL of water, and the resulting suspension was stirred at room temperature for 1 h. The solid was collected by vacuum filtration, washed with copious amount of water, followed by methanol, and then air-dried. The crude product was checked on TLC and ^{1}H NMR to conform

the isomer presence and it is found to be around 45:55 ratio based on ^{1}H NMR spectra. The crude mixture was purified on chromatography using 3% ethyl acetate in hexane as an eluent, and the solvent was removed under vacuum to obtain a pure product. Yield 40%. ^{1}H NMR (300 MHz, $CDCl_3$, ppm) δ = 8.92 (d, 2 H, H_{Th}), 7.64 (d, 2 H, H_{Th}), 7.27 (m, 2 H, H_{Th}), 4.07 (d, 4 H, *N*-CH_2), 1.66–1.74 (m, 4 H, *N*-CH_2-CH_2), 1.41–1.28 (m, 12 H), 0.87 (t, 12 H). ^{13}C NMR (125 MHz, $CDCl_3$, ppm) δ = 161.37, 140.02, 135.21, 130.63, 129.77, 128.58, 107.70, 42.21, 31.39, 29.89, 26.53, 22.53, 13.98. MALDI-TOF, Mw 468.9518. Anal. Calcd for $C_{26}H_{32}N_2O_2S_2$: C, 66.63; H, 6.88; N, 5.98; S, 13.68. Found: C, 66.83; H, 7.01; N, 6.16; S, 13.42.

3,6-Bis(5-bromothiophen-2-yl)-2,5-dihexyl-2,5-dihydropyrrolo[3,4-c]pyrrole-1,4-dione (DPP^{Th}-Br_2). Compound DPP^{Th}-Br_2 was synthesized by following a modified procedure reported.[5] To a 100 mL two-neck round bottom flask, compound DPP^{Th} (0.60 g, 1.28 mmol) was added and dissolved in to dry DMF (10 mL) under nitrogen atmosphere. The reaction mixture was cooled down to 0 °C in ice bath and *N*-bromosuccinimide (NBS) (0.46 g, 2.6 mmol) was added portion wise. It was stirred at room temperature for 12 h. This mixture was poured into water (100 mL) and stirred for 1 h. Solid was vacuum filtered and washed with MeOH and H_2O to remove excess NBS. The crude solid was purified through column chromatography using hexane:DCM (1:1) as an eluent. Compound DPP^{Th}-Br_2 was obtained as a dark purple solid (0.58 g, yield 83%). ^{1}H NMR (300 MHz, CD_2Cl_2, ppm) δ = 8.67 (d, 2 H, H_{Th}), 7.27 (d, 2 H, H_{Th}), 3.98 (d, 4 H, *N*-CH_2), 1.64–1.74 (m, 4 H, *N*-CH_2-CH_2), 1.30-1.40 (m, 12 H), 0.89 (t, 6 H). ^{13}C NMR (75 MHz, $CDCl_3$, ppm) δ = 160.95, 139.01, 135.32, 131.62, 131.12, 119.10, 107.90, 42.30, 31.90, 29.95, 26.80, 22.63, 14.07. UV/Vis (toluene): λ_{max}: 550 nm. MALDI-TOF, Mw 626.47 (626.55). Anal. Calcd for $C2_6H_{30}Br_2N_2O_2S_2$: C, 49.85; H, 4.83; Br, 25.51; N, 4.47; O, 5.11; S, 10.24. Found: C, 49.76; H, 4.75; Br, 26.05; N, 4.55; S, 9.89.

3,6-Bis(5-(4,4,5,5-tetramethyl-4,5-dihydro-3-oxid-1-oxyl-1 H-imidazol-2-yl)thiophene-2yl)-2,5-dihexyl-2,5-dihydropyrrolo[3,4-c]pyrrole-1,4-dione (DPP^{Th}-NN_2). A solution of dibromide DPP^{Th}-Br_2 (100 mg, 0.16 mmol), nitronyl nitroxide gold complex (218 mg, 0.35 mmol), and [$Pd(PPh_3)_4$] (20 mg) in THF (15 mL) was stirred at 65 °C in an argon atmosphere for 16 h. The reaction mixture was cooled rt and solvent was evaporated. The residue was purified by column chromatography on silica gel by using CH_2Cl_2/ethyl acetate (10:1) solvent mixture as an eluent. The resulted product was recrystallized two times from a mixture of CH_2Cl_2 with MeOH to obtain diradical DPP^{Th}-NN_2 (90 mg, 72 %). Compound DPP^{Th}-NN_2 was eluted with R_f = 0.7 (10:1 mixture of CH_2Cl_2/ethyl acetate); IR: υ=2956, 2927, 2856, 1662, 1552, 1436, 1400, 1367, 1367, 1211, 1176, 1134, 1095, 870, 827, 729, 620, 540 cm^{-1}; UV/Vis (toluene): λ_{max}: two peaks at 573 and 624 nm and a shoulder at ~690 nm; MALDI-TOF MS: *m*/*z* calcd. (%) for $C_{52}H_{76}N_4O_4S_4$:

778.35 (100) $[M]^+$, 779.36 (43) $[M+1]^+$; found: 778 (average M_W); elemental analysis calcd.: C 61.67, H 6.99, N 10.79, S 8.23; found: C 61.80, H 7.1, N 10.3, S 8.05. Melting point, 216 °C.

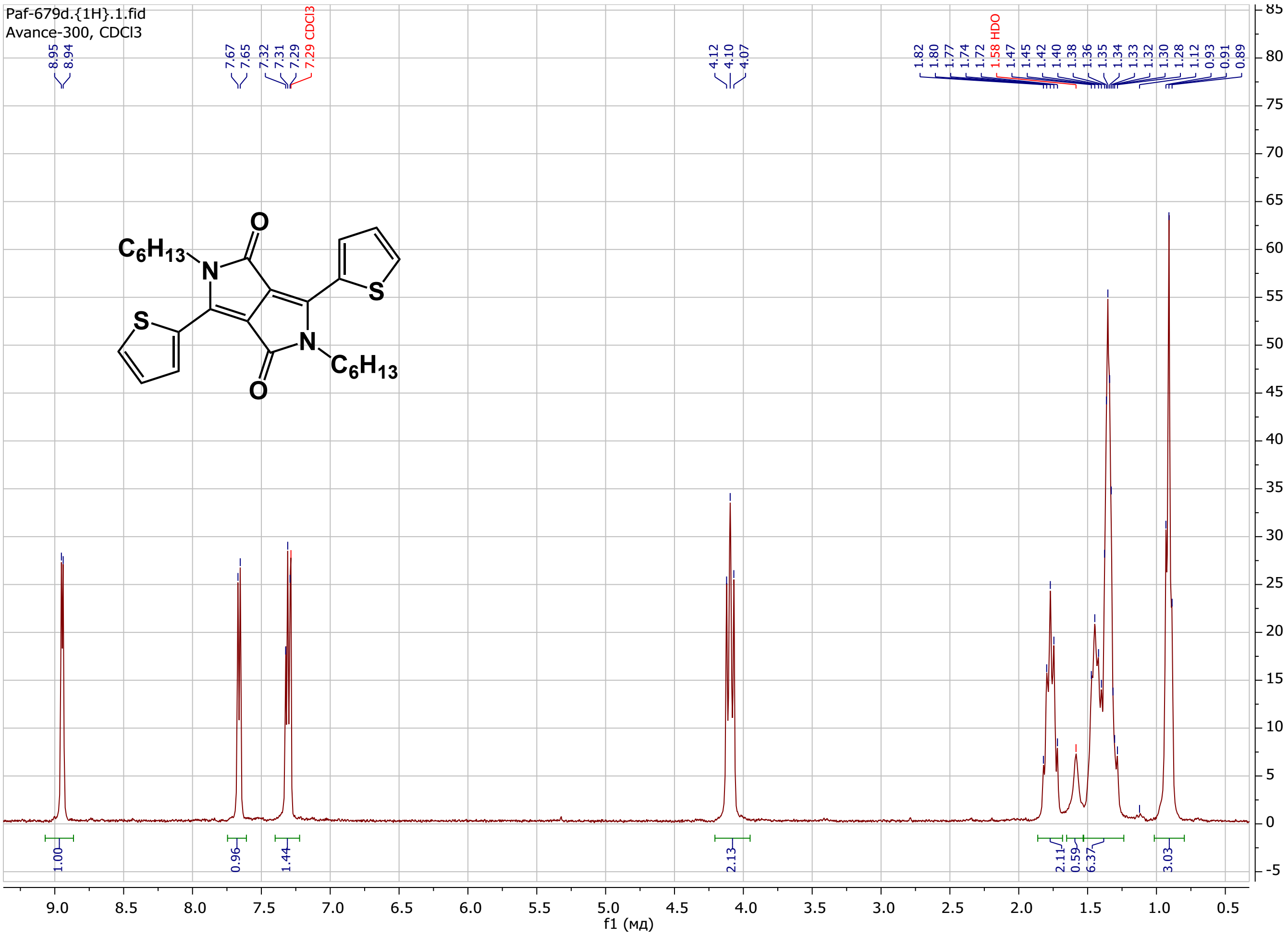

Figure S1. ^{1}H NMR spectrum of DPP^{Th} ($CDCl_3$, 300 MHz).

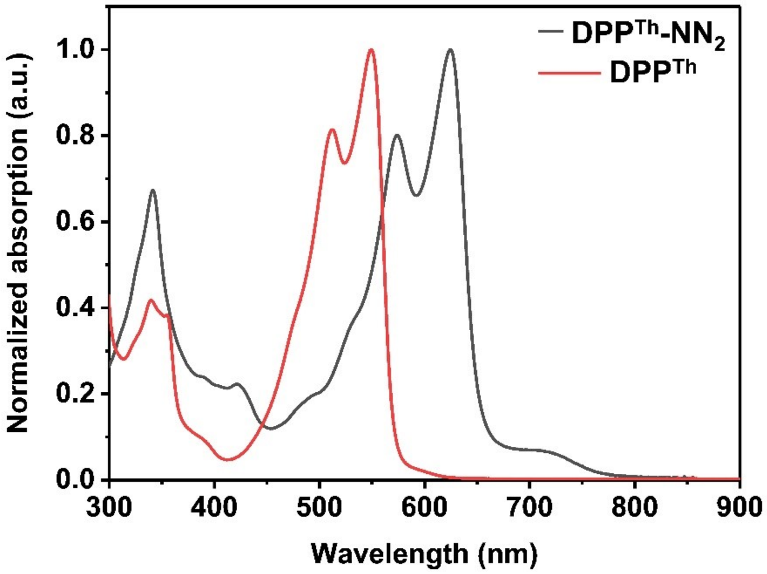

Figure S2. Normalized UV-vis absorption spectrum of DPPTh and DPPTh-NN_2 in toluene at room temperature.

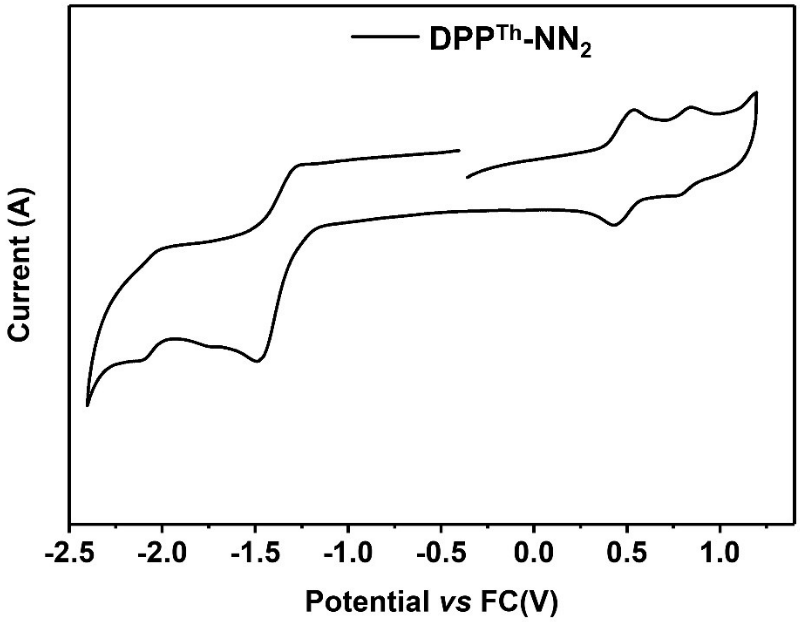

Figure S3. Cyclic voltammograms of DPPTh-NN_2 in 0.1M Bu_4NPF_6 in dichloromethane.

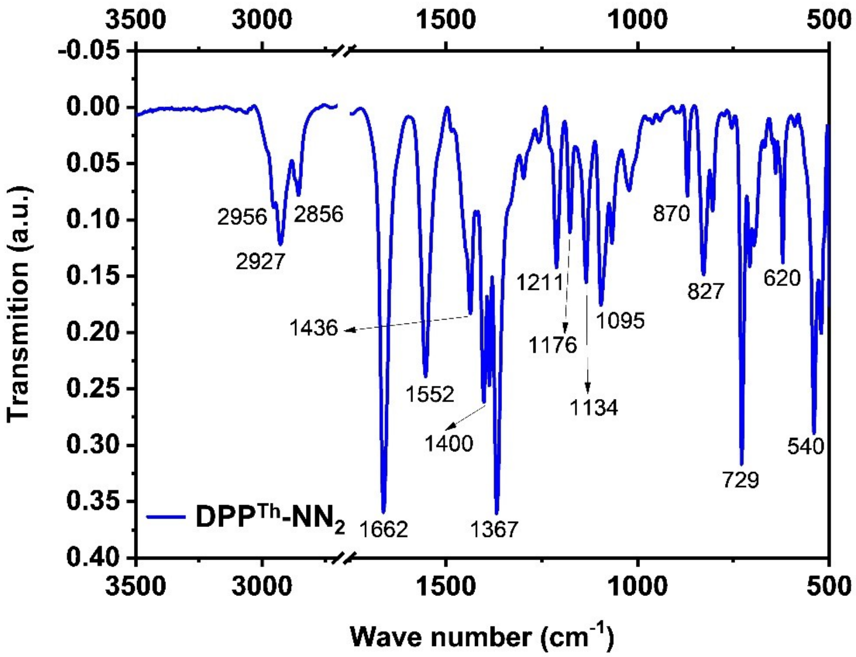

Figure S4. IR spectrum of DPP^{Th}-NN_2 diradical.

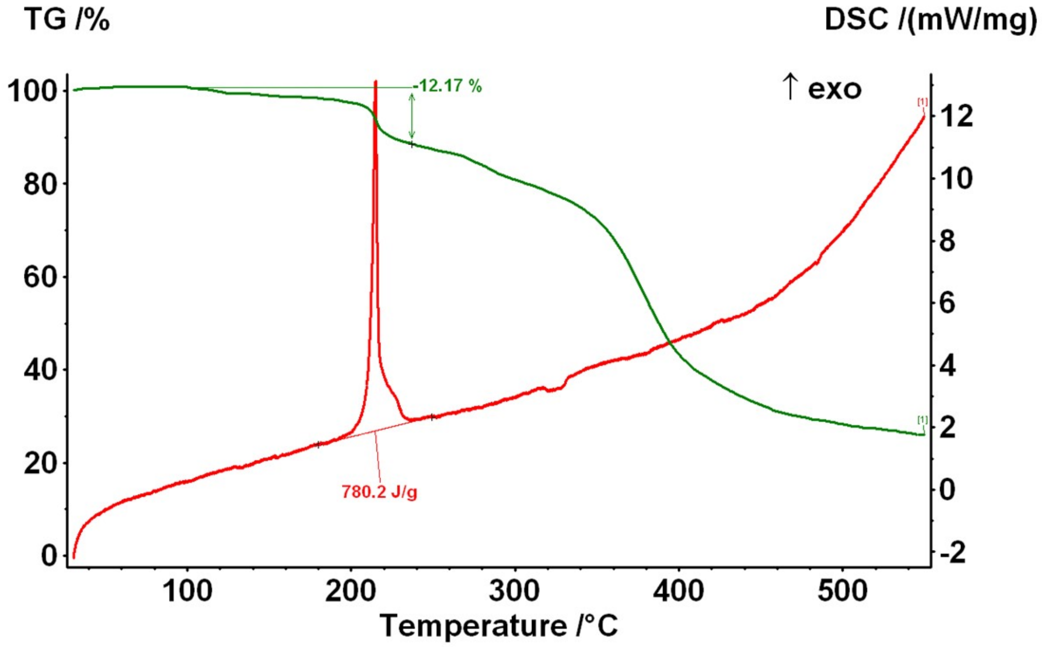

Figure S5. Thermogravimetric analysis (red) and differential scanning calorimetry (green) curves of DPP^{Th}-NN_2.

Table S1. Bond Length Data from Single Crystal X-ray Diffraction for DPP^{Th}-NN_2.

Bond	Length, Å	Bond	Length, Å
C1A—O21A	1.238 (17)	C1B—O21B	1.258 (17)
C1A—N4Ai	1.457 (19)	C1B—N4Bii	1.418 (17)
C1A—C2A	1.47 (2)	C1B—C2B	1.46 (2)
C2A—C3A	1.36 (2)	C2B—C3B	1.397 (18)
C2A—C2Ai	1.38 (3)	C2B—C2Bii	1.39 (3)
C3A—N4A	1.402 (18)	C3B—N4B	1.355 (18)
C3A—C5A	1.48 (2)	C3B—C5B	1.47 (2)
N4A—C22A	1.414 (18)	N4B—C22B	1.446 (16)
N4A—C1Ai	1.457 (19)	N4B—C1Bii	1.418 (17)
C5A—C6A	1.406 (19)	C5B—C6B	1.395 (19)
C5A—S9A	1.694 (17)	C5B—S9B	1.712 (13)
C6A—C7A	1.426 (19)	C6B—C7B	1.423 (19)
C7A—C8A	1.348 (19)	C7B—C8B	1.371 (18)
C8A—C10A	1.43 (2)	C8B—C10B	1.47 (2)
C8A—S9A	1.763 (14)	C8B—S9B	1.694 (16)
C10A—N14A	1.309 (19)	C10B—N14B	1.338 (17)
C10A—N11A	1.347 (18)	C10B—N11B	1.335 (18)
N11A—O19A	1.284 (14)	N11B—O19B	1.274 (14)
N11A—C12A	1.476 (18)	N11B—C12B	1.523 (16)
C12A—C13A	1.558 (19)	C12B—C13B	1.514 (19)
C12A—C16A	1.559 (18)	C12B—C16B	1.573 (17)
C12A—C15A	1.57 (2)	C12B—C15B	1.526 (19)
C13A—C17A	1.517 (19)	C13B—C17B	1.571 (17)
C13A—C18A	1.52 (2)	C13B—C18B	1.526 (18)
C13A—N14A	1.528 (19)	C13B—N14B	1.530 (17)
N14A—O20A	1.310 (15)	N14B—O20B	1.285 (14)
C22A—C23A	1.522 (18)	C22B—C23B	1.500 (17)
C23A—C24A	1.56 (2)	C23B—C24C	1.38 (4)
C24A—C25A	1.50 (2)	C23B—C24B	1.69 (5)
C25A—C26A	1.43 (2)	C24B—C25B	1.45 (6)
C26A—C27A	1.49 (2)	C25B—C26B	1.52 (7)
		C26B—C27B	1.72 (5)
		C24C—C25C	1.54 (4)
		C25C—C26C	1.57 (4)
		C26C—C27B	1.46 (4)

2. Photo induced EPR-experiments

The compound DPP^{Th}-NN_2 was dissolved in toluene in concentration of approximately 0.5 mM. Pulse EPR experiments were done using commercial Bruker Elexsys E580 spectrometer equipped with liquid He temperature control system. All measurements were carried out at X-band (~9.7 GHz) and at 10 K. Nd:YaG laser system LOTIS-TII was used for photoexcitation at 532 nm (second harmonic) with 10 Hz repetition rate and power of 20-40 mJ per pulse. In all cases, we recorded echo-detected (ED) EPR spectra using Hahn echo sequence $\pi/2$ - τ - π - τ - echo with 20/40 ns pulse lengths and τ=200 ns. In the case of photoexcitation applied, the laser pulse preceded the sequence by ca. 500 ns.

3. Electronic structure of the DPPTh-NN$_2$ diradical

3.1. Quantum chemical calculations

All quantum chemical calculations were performed for model structures that differ from the structures of the compounds under study by replacing the long n-butyl substituents with methyl groups. This substitution should only slightly affect the electronic and spectral properties of the parent DPPTh and the DPPTh-NN$_2$ biradical. Moreover, two types of model structures were used in calculations: the model geometry of DPPTh-NN$_2$ diradicals and their pairs obtained from XRD analysis or the model geometry optimized in toluene solution at the B3LYP/def2-TZVP level of theory;[4] the solvent was taken into account according to the CPCM model.[5]

The parameters of the intramolecular exchange interaction ($H = -2J\hat{S}_1\hat{S}_2$) were computed using the accurate *ab initio* CASSCF[6] and CASSCF/NEVPT2[7] procedures. To calculate the J parameters for intermolecular exchange interactions, the spin-unrestricted broken-symmetry (BS) approach[8] at the BS-B3LYP/def2-TZVP level of theory was utilized using the Yamaguchi formula[9]

$$J = -\frac{E^{HS} - E^{LS}_{BS}}{\langle S^2\rangle^{HS} - \langle S^2\rangle^{LS}_{BS}}.$$

3.2. Calculations using the model geometry based on the XRD structure

It can be seen from Figure S6 that the conjugation of the NN radical fragments with the DPPTh core leads to a noticeable delocalization of the spin density from NN fragments to the thiophene rings.

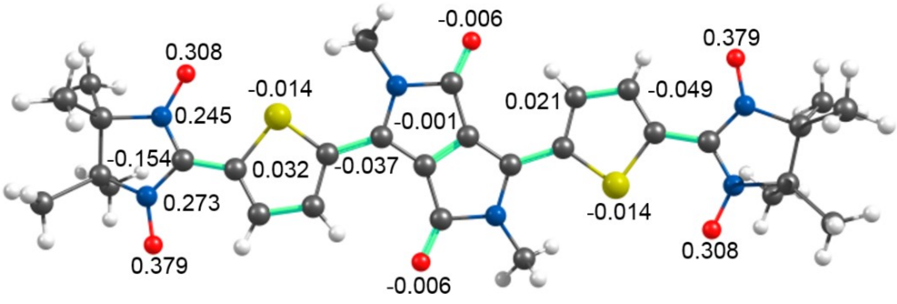

Figure S6. The atomic spin population in the triplet state of the DPPTh-NN$_2$ calculated at the UB3LYP/def2-TZVP level at a model geometry based on the XRD structure for diradical A.

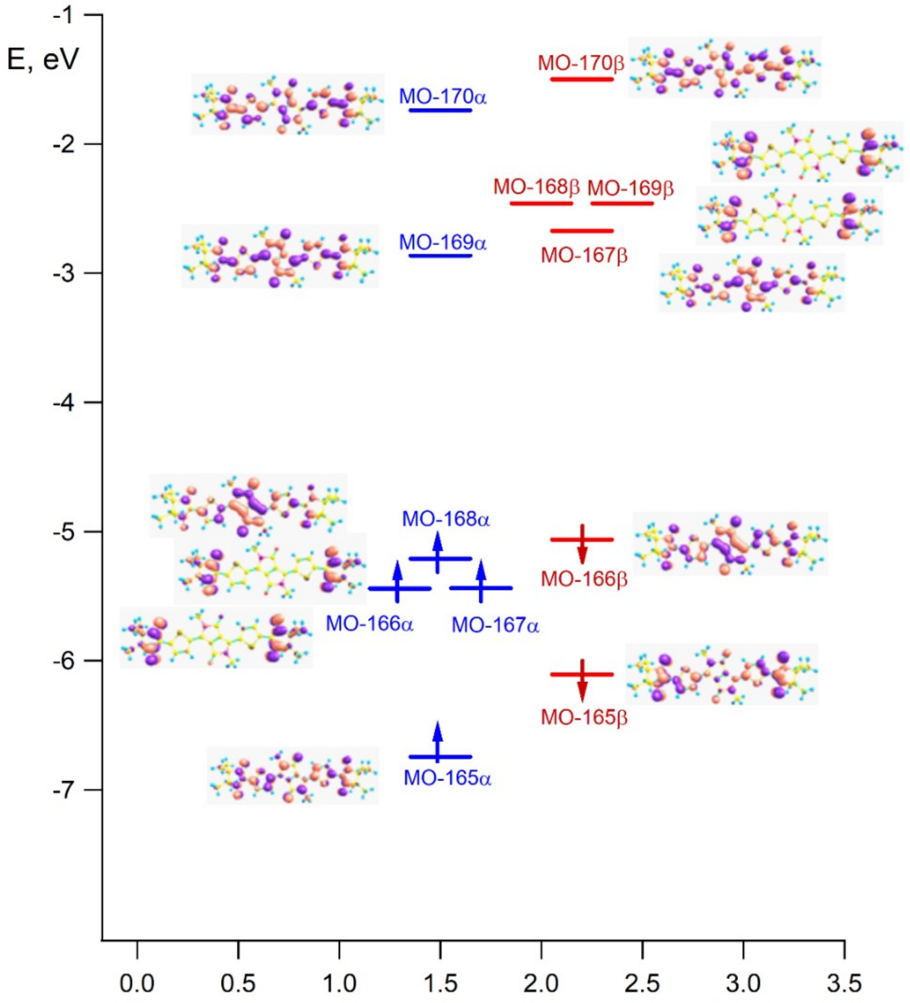

Figure S7. A number of highest occupied and lowest unoccupied molecular orbitals (MOs) of α-(blue energy levels) and β-types (red energy levels) of the DPPTh-NN_2 diradical in the high-spin triplet state calculated at the UB3LYP/def2-TZVP level.

We demonstrated previously[10,11] that BS-DFT calculations overestimate significantly parameters of the intramolecular exchange interaction for the diradicals, the SOMOs (or non-bonding molecular orbitals – NBMOs) of which can be localized in different regions of the molecule (disjoint type SOMOs). MO diagram of Figure S7, demonstrates that SOMOs of the DPPTh-NN_2 diradical are degenerate and localized on the NN fragments. Wherein, if to take the sum and difference of these SOMOs (MO-166α and MO-167α), new SOMOs localized on different NN fragments will be obtained, it's just the disjoint type SOMOs.

Thus, the parameters J of the exchange interaction between NN fragments of the DPP^{Th}-NN_2 diradical were calculated using the accurate CASSCF and CASSCF/NEVPT2 approaches. Calculations were carried out for two model geometries: obtained on the basis of XRD analysis and optimized in toluene. First, the calculations were performed taking into account only the lowest triplet and singlet states of the diradical. In additions, the energies of six lower states were

calculated, namely, four more states (one singlet, two triplets and quintet) were added to the previous singlet and triplet, resulting from the exchange interaction of the lowest excited triplet state with doublet states of NN fragments. The largest active space used in these calculations consisted of 14 electrons on 13 MOs. Figure S8 displays the isosurfaces of the corresponding active MOs, as well as their average occupation numbers and energies estimated according to the ORCA protocol.

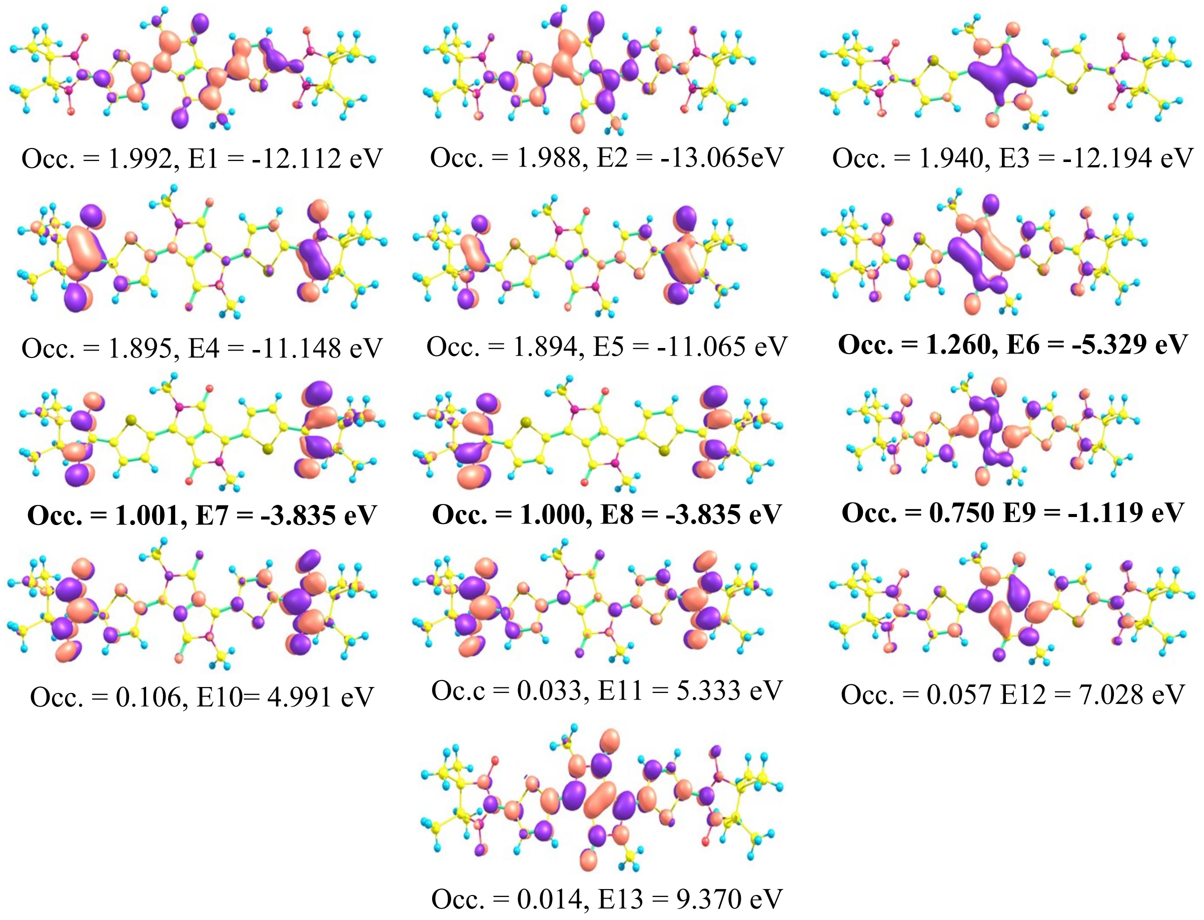

Figure S8. Molecular orbitals (iso-surfaces at 0.03 level) of the active space, consisting of 14 electrons on 13 MOs, used to calculate the energies of the six lower multiplets of the DPP^{Th}-NN_2 diradical, as well as the energies and averaged occupation numbers of these MO.

Intermolecular exchange interactions.

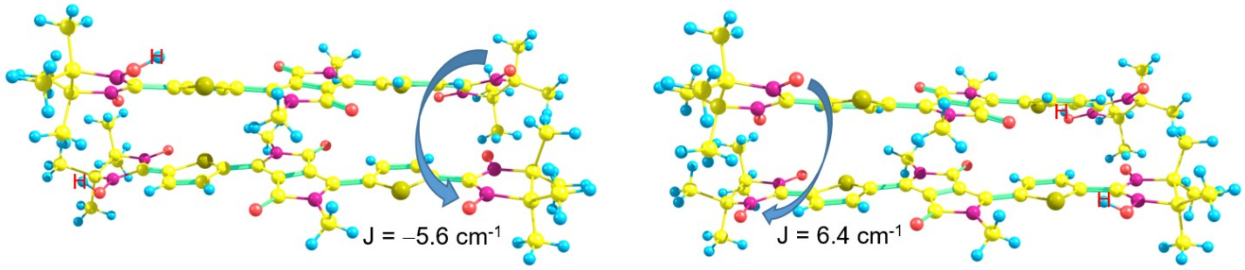

Figure S9. Pairs of diradicals extracted from the XRD structure and modified by adding 2 hydrogen atoms to NO groups of two neighboring radical fragments and predicted at the BS-UB3LYP level values of parameter *J* for remaining radical fragments.

To calculate the parameters of intermolecular exchange interactions, we used the BS-DFT approach, since in such cases it works correctly.[12] The calculations were carried out for pairs of diradicals in a stack in which we turned two adjacent radical fragments into diamagnetic ones by replacing the NO fragment with NOH (Figure S9). For two pairs, parameters *J* different in sign were predicted: –5.6 and 6.4 cm^{-1}. An antiferromagnetic exchange interaction was predicted in the case when ONCNO fragments of neighboring radical centers are almost parallel, and a ferromagnetic one, in the case of a significant deviation from parallel arrangement of the corresponding fragments.

The energy diagrams for six lower energy levels.

The energy diagrams for the DPP^{Th}-NN_2 diradical obtained using both model geometries are shown in Figure S10. Figure S10 and Table 1 of the main text show that in both cases the quintet state is the lowest energy excited state, indicating a ferromagnetic interaction between the local triplet state and doublets of both NN fragments. It can also be seen that the splitting of energy levels noticeably increases, and the energy of the quintet state decreases significantly when using the optimized geometry. This indicates an increase in the ferromagnetic interaction for the optimized geometry. Most likely this is due to planarization of the molecular backbone during optimization. To our knowledge, this is the first time such calculations have been carried out for a dye substituted by two radical fragments.

According to the CASSCF(14,13) calculations, the main contribution (79.4%) to the wavefunction of the Q_1 state is made by the configuration in which five MOs (Fig. S8, E1 – E5) are occupied twice, on four MOs (E6 – E9) there are one electron each, and all other MOs (E10 – E13) are empty. Comparison with MOs presented in Figure S7 demonstrates that MOs 7 and 8 are localized on NN fragments and are similar to SOMOs 166α and 167α. In turn, singly-occupied

active MOs 6 and 9 (E6, E9) are very similar to HOMO (MO-168α) and LUMO (MO-169α), the transitions of electron between which make the main contribution to the most intense band in the UV-Vis spectrum of the DPPTh-NN$_2$ diradical (Figs 4 and 5 of main text). Thus, the quintet state Q_1, as well as S_1, T_2 and T_3, presented in Figure S10, arose as a result of the exchange interaction of the triplet state, corresponding to the HOMO → LUMO excitation and localized predominantly on the DPPTh core, with doublets of NN moieties.

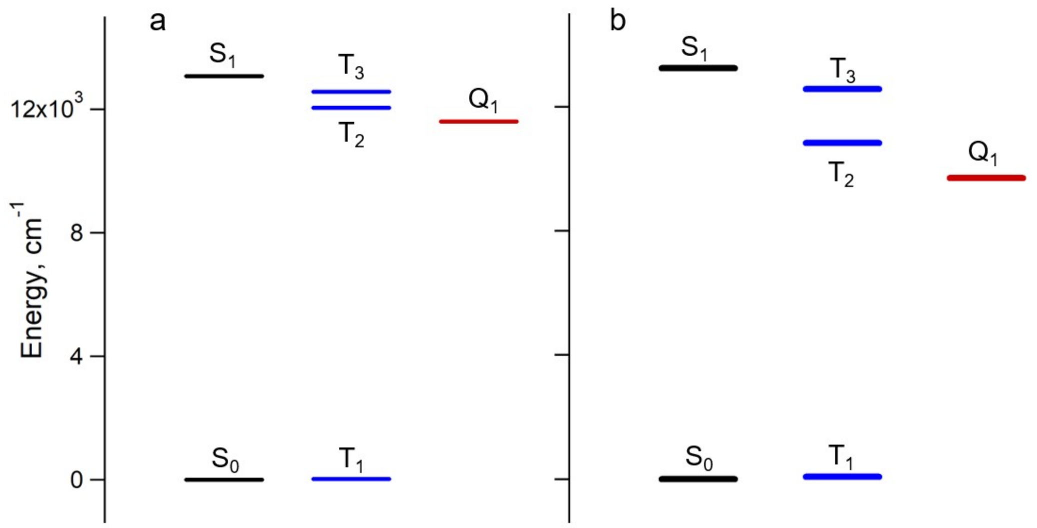

Figure S10. Energy diagrams obtained from SA-CASSCF(14,13)/NEVPT2/def2-TZVP calculations taking into account the six lowest energy states of the DPPTh-NN$_2$ diradical with model geometries based on XRD analysis (a) and optimized at the UB3LYP/def2-TZVP (b).

It is reasonable to assume that the relative energies of the S_1, T_2, T_3 and Q_1 states are determined by the spin Hamiltonian of the form

$$\hat{H} = -2J_1(\hat{S}_{DPP^{Th}} \cdot \hat{S}_1 + \hat{S}_{DPP^{Th}} \cdot \hat{S}_2) - 2J_2(\hat{S}_1 \cdot \hat{S}_2),$$

where parameter J_1 of the exchange interaction between the local triplet state and both doublets of NN groups were proposed to be the same. The energies for this spin Hamiltonian are defined by the matrix equation

$$\begin{pmatrix} S_1 \\ T_2 \\ Q_1 \\ T_3 \end{pmatrix} = \begin{pmatrix} 4 & -1/2 \\ 2 & -1/2 \\ -2 & -1/2 \\ 0 & 3/2 \end{pmatrix} \begin{pmatrix} J_1 \\ J_2 \end{pmatrix}.$$

The two exchange interaction parameters (J_1, J_2) can be extracted by calculating the pseudo-inverse matrix and choosing the order of the levels T_2 and T_3 leading to physically reasonable values of J_1 and J_2.

The values of these parameters, together with the parameter J for the diradical in the ground state, are presented in the Table 1 (see main text). Table 1 shows that the parameters of the exchange interaction between radical fragments NN (J and J_2) are small and negative for both states (the ground state, J, and the excited state, J_2). It can also be seen that these parameters increase markedly in absolute value for the optimized geometry. As regards the parameter of the exchange interaction between the triplet of the DPP^{Th} core and NN fragment (J_1), it is positive and very large, and it increases significantly for the optimized geometry (from 244 to 588 cm^{-1}).

Since the Q_1 state is the lowest energy state among the quintets and is also a high-spin state, its electronic structure can be calculated at the DFT level; the same is true for T_1 state. Figure S10 shows the atomic spin populations calculated at the UB3LYP level for the T_1 and Q_1 multiplets. Moreover, the zero-field splitting parameters can be calculated for them at the RO-DFT level (Table 1 of main text).

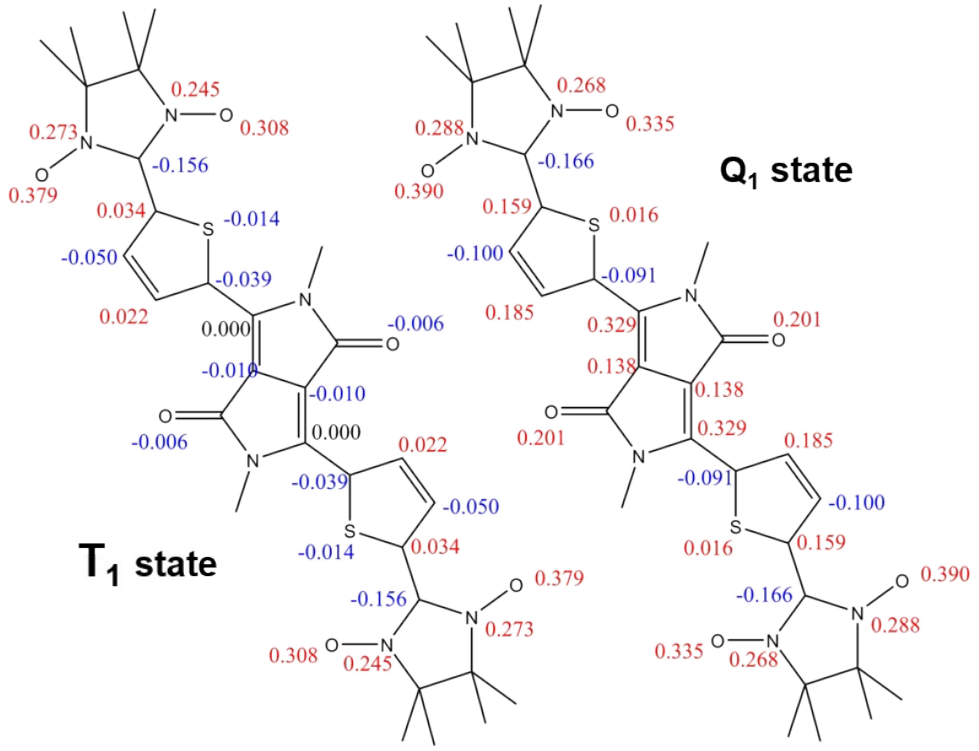

Figure S11. Atomic spin populations calculated at the UB3LYP/def2-TZVP level for the T_1 and Q_1 multiplets.

References

1 M. Fukuda, K. Kodama, H. Yamamoto and K. Mito, *Dyes Pigm.*, 2004, **63**, 115.

2 A. B. Tamayo, M. Tantiwiwat, B. Walker and T.-Q. Nguyen, *J. Phys. Chem. C*, 2008**, 112**, 15543.

3 A. B. Tamayo, B. Walker and T.-Q. Nguyen, *J. Phys. Chem. C*, 2008**, 112**, 11545.

4 a) A. D. Becke, *J. Chem. Phys.*, 1993, **98**, 5648; b) C. Lee, W. Yang and R. G. Parr, *Phys. Rev. B*, 1988, **37**, 785; c) F. Weigend, and R. Ahlrichs, *Phys. Chem. Chem. Phys*., 2005, **7**, 3297.

5 V. Barone and M. Cossi, *J. Phys. Chem. A*, 1998, **102**, 1995.

6 M. Frisch, I. N. Ragazos, M. A. Robb and H. B. Schlegel, *Chem. Phys. Lett.*, 1992, **189**, 524.

7 a) K. Andersson, P.-Å. Malmqvist and B. O. Roos, *J. Chem. Phys.*, 1992, **96**, 1218; b) C. Angeli, R. Cimiraglia, S. Evangelisti, T. Leininger and J. P. Malrieu, *J. Chem. Phys.* 2001, **114**, 10252.

8 H. Nagao, M. Nishino, Y. Shigeta, T. Soda, Y. Kitagawa, T. Onishi, Y. Yoshika and K. Yamaguchi, *Coord. Chem. Rev*., 2000, **198**, 265.

9 T. Soda, Y. Kitagawa, T. Onishi, H. Nagao, Y. Yoshioka and K. Yamaguchi, *Chem. Phys. Lett*., 2000, **319**, 223.

10 E. V. Tretyakov, V. I. Ovcharenko, A. O. Terent′ev, I. B. Krylov, T. V. Magdesieva, D. G. Mazhukin and N. P. Gritsan, *Russ. Chem. Rev*., 2022, **91**, RCR5025.

11 E. Tretyakov, A. Keerthi, M. Baumgarten, S. Veber, M. Fedin, D. Gorbunov, I. Shundrina and N. Gritsan, *ChemistryOpen*, 2017, **6**, 642.
